# A Genetically Engineered Reporter System Designed for ^2^H-MRI Allows Quantitative *In Vivo* Mapping of Transgene Expression

**DOI:** 10.1021/jacs.4c09572

**Published:** 2024-11-11

**Authors:** Hyla Allouche-Arnon, Elton T. Montrazi, Balamurugan Subramani, Michal Fisler, Inbal Spigel, Lucio Frydman, Tevie Mehlman, Alexander Brandis, Talia Harris, Amnon Bar-Shir

**Affiliations:** † Department of Molecular Chemistry and Materials Science, Weizmann Institute of Science, Rehovot 7610001, Israel; ‡ Department of Chemical and Biological Physics, Weizmann Institute of Science, Rehovot 7610001, Israel; § Department of Life Sciences Core Facilities, Weizmann Institute of Science, Rehovot 7610001, Israel; ∥ Department of Chemical Research Support, Weizmann Institute of Science, Rehovot 7610001, Israel

## Abstract

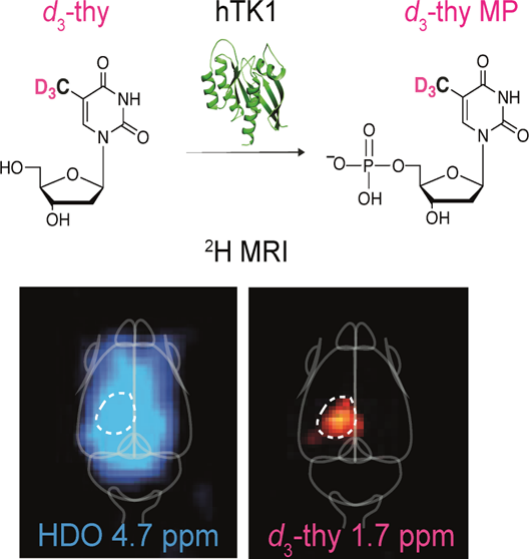

The ability to obtain quantitative spatial information on subcellular processes of deep tissues *in vivo* has been a long-standing challenge for molecular magnetic resonance imaging (MRI) approaches. This challenge remains even more so for quantifying readouts of genetically engineered MRI reporters. Here, we set to overcome this challenge with a molecular system designed to obtain quantitative ^2^H-MRI maps of a gene reporter. To this end, we synthesized deuterated thymidine, *d*_3_-thy, with three magnetically equivalent deuterons at its methyl group (-CD_3_), showing a singlet peak with a characteristic ^2^H-NMR frequency (δ = 1.7 ppm). The upfield 3.0 ppm offset from the chemical shift of the HDO signal (δ = 4.7 ppm) allows for spectrally resolving the two ^2^H NMR signals and quantifying the concentration of *d*_3_-thy based on the known concentration of a tissue’s HDO. Following systemic administration of *d*_3_-thy, its accumulation as *d*_3_-thy monophosphate in cells expressing the human thymidine kinase 1 (hTK1) transgene was mapped with ^2^H-MRI. The data obtained *in vivo* show the ability to use the *d*_3_-thy/hTK1 pair as a reporter probe/reporter gene system to quantitatively map transgene expression with MRI. Relying on a structurally unmodified reporter probe (*d*_3_-thy) to image the expression of unmutated human protein (hTK1) shows the potential of molecular imaging with ^2^H-MRI to monitor gene reporters and other relevant biological targets.

## Introduction

*In vivo* mapping of gene expression using genetically engineered heterologous reporters has been a long-standing challenge for the biological sciences. Among the imaging modalities that allow spatial visualization of transgene expression with unlimited depth penetration through a living subject, magnetic resonance imaging (MRI) stands out.^1,2^ Genetically engineered reporters for MRI can be classified into two main types. In one type, heterologous reporter proteins are expressed and directly monitored through their ability to modulate a tissue’s MRI signal. Iron storage proteins are used as MRI reporter genes using T_2_ and T_2_* contrast mechanisms to map the transgene expression *in vivo*.^3,4^ Polypeptides and proteins rich with lysine and arginine residues are used as heterologous reporters based on their ability to modulate the chemical exchange saturation transfer (CEST) MRI contrast.^5−7^ Expressing aquaporin to modulate intracellular water diffusivity, and thus, diffusion-weighted MRI, showed the ability to use water channels as MRI reporter genes.^8^ Similarly, urea transporters can increase the transmembrane water exchange rates toward MRI signal attenuation.^9^ The commonality of reporter proteins of this type is that they do not require additional imaging agents and are advantageous when this requirement is restricted. Nevertheless, this type of MRI reporter gene relies on high expression levels to obtain reliable MRI signals, does not allow the removal of ^1^H-MR background signals, and the ability to quantify their expression levels based on their MRI readouts remained a challenge.

An alternative type of genetically engineered MRI reporters is the one based on reporter-protein/reporter-probe pairs. In these systems, the expression of the transgene is spatially mapped after the administration of an imageable reporter probe, which is accumulated (or converted) solely in cells expressing the reporter protein.^10,11^ One very sensitive reporter system is based on the organic anion-transporting polypeptide (oatp1a1 or oatp1b3), which can effectively transport a Gd-based MRI reporter probe to produce intense T_1_-weighted MR images of cells expressing an oatp1 reporter gene *in vivo*.^12−14^ Another example is the divalent metal ion transporter (DMT1), which can transport the paramagnetic Mn^2+^ to enhance the MRI contrast tissues expressing the transgene.^15^ The emergence of CEST MRI stimulated the use of nonmetallic reporter probes as imageable agents of MRI reporter genes.^16^ Synthetic analogs of thymidine and deoxy-cytidine were used as CEST reporter probes for *in vivo* MRI mapping of the expression of the herpes simplex virus type-1 thymidine kinase (HSV1-TK) and the *Drosophila melanogaster* deoxyribonucleoside kinase (*Dm*-dNK) transgenes, following the introduced probe phosphorylation and accumulation.^17−20^ Nevertheless, these genetically engineered reporter-protein/reporter-probe systems are all based on ^1^H-MRI; thereby, they experience large background signals, which requires acquiring a preinjection MR image to quantify the absolute concentration of the accumulated reporter probes. Moreover, relying on synthetically modified molecular probes limits their enzymatic conversion by various putative native reporter genes that can be proposed to map gene expression with MRI.

The recent emergence of ^2^H-MRI strategies,^21,22^ primarily for metabolic imaging, opened opportunities to use deuterium-labeled natural molecules (e.g., glucose,^21,22^ pyruvic acid,^23^ choline,^24^ acetate,^25^ fumarate,^26^ and others^27^) for molecular MR imaging studies. The relatively short T_1_ relaxation times of ^2^H—ranging from a few 10s to a few 100s of milliseconds—allow one to compensate in part for the fact that the gyromagnetic ratio of ^2^H is ∼6.5 times lower than that of ^1^H, by using rapid repetitive scans for signal averaging. Moreover, unique to ^2^H-MRI and very attractive for molecular MRI, is the ability to obtain quantitative maps of the administered ^2^H-labeled probe, based on utilizing the endogenous signal of HDO (∼11 mM in biological tissues) as an internal reference for ^2^H-MR signal quantification.^22^ This practice does not apply to genetically engineered reporter systems developed for other non-^1^H-nuclei such as ^19^F,^28^ which, although perhaps preferable sensitivity- and resolution-wise, require an external reference compound for their MR signal quantification. We present here the design and implementation of a genetically engineered reporter system that exploits all these unique features for mapping and quantifying the expression of a reporter gene with ^2^H-MRI. To this end, ^2^H-labeled thymidine (*d*_3_-thy) was synthesized as a deuterated reporter probe, to image the expression of human thymidine kinase-1 (hTK1) as a reporter gene. *d*_3_-thy facilitates cellular transporters, followed by its phosphorylation by cytosolic hTK1 to obtain a *d*_3_-thy-monophosphate (*d*_3_-thy-MP) that accumulates in cells, giving rise to a quantifiable ^2^H-MRI signal solely in cells expressing the transgene.

## Results and Discussion

### Synthesis and Characterization of *d*_3_-thy

**Scheme 1:**
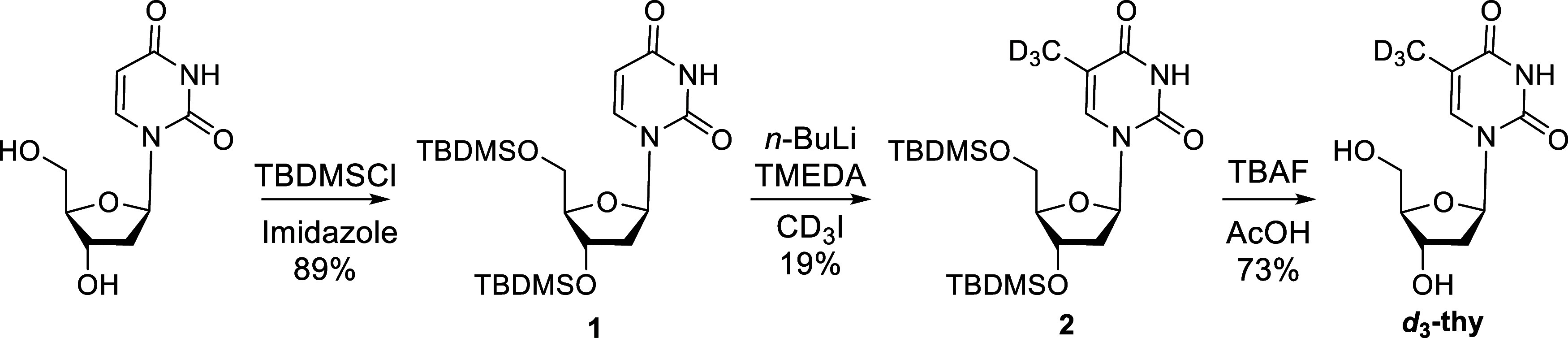
Synthesis of *d*_3_-thy

Deuterated thymidine (*d*_3_-thy) was synthesized by methylation of a silyl-protected 2′-deoxyuridine with a CD_3_I, as shown in Scheme 1, after optimization of a previously proposed synthetic protocol (Supporting Information).^29^

**Figure 1:**
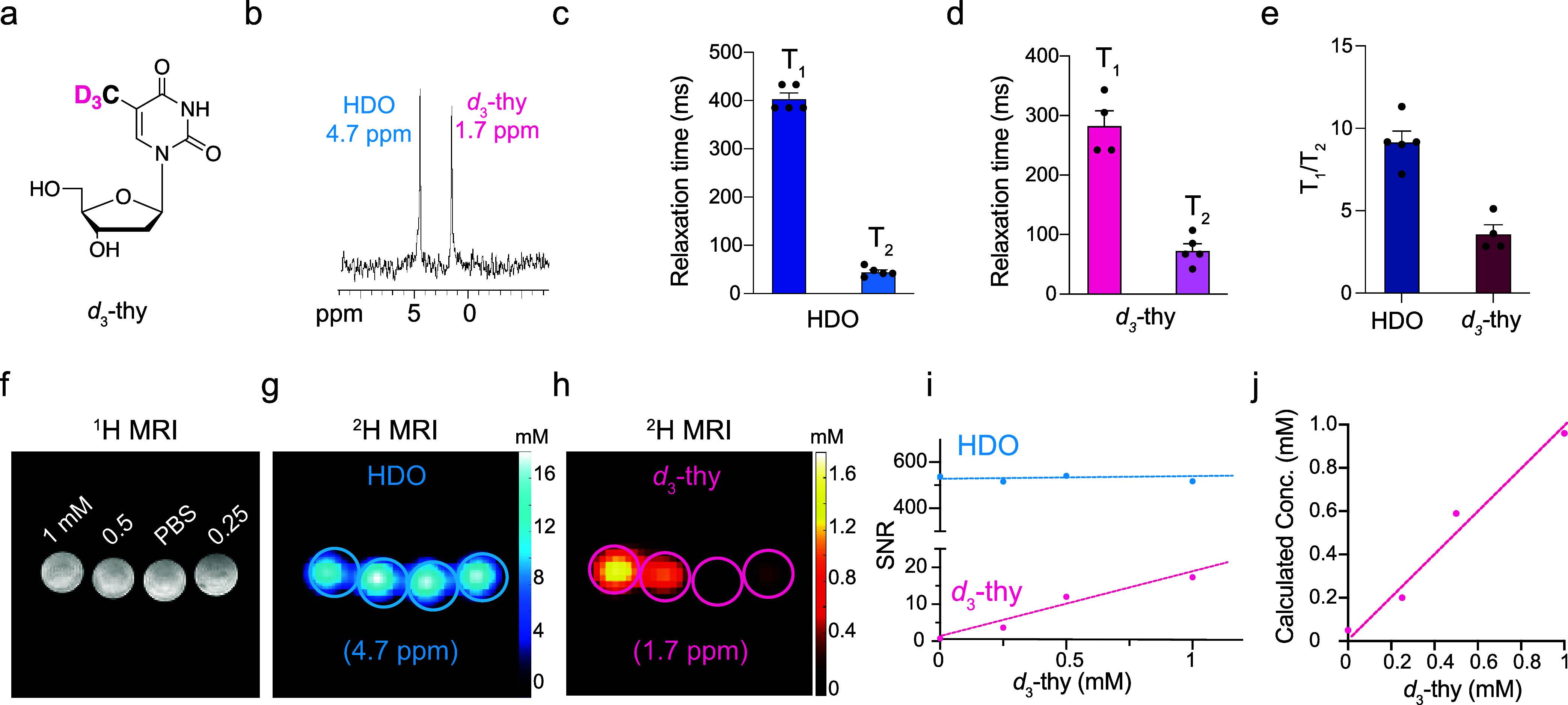
^2^H-MR properties of deuterated thymidine (*d*_3_-thy). (a) Chemical structure of *d*_3_-thy, with the methyl deuterons used for ^2^H-MR studies shown in pink. (b) ^2^H-NMR spectra of 5 mM *d*_3_-thy in PBS, resolving two singlet peaks, one of HDO at δ = 4.7 ppm and another of *d*_3_-thy methyl moiety at δ = 1.7 ppm. T_1_ and T_2_ relaxation times of the HDO deuteron (c) and the *d*_3_-thy deuterons (d) evaluated for a 5 mM *d*_3_-thy in 4% (weight) agarose solution, measured on a 14.1 T NMR spectrometer. (e) T_1_/T_2_ ratio of HDO and *d*_3_-thy as calculated from (c) and (d). (f-h) Phantom MRI experiments of PBS solutions without *d*_3_-thy or with variable concentrations of *d*_3_-thy (0.25, 0.5, and 1 mM) performed on a 15.2 T MRI showing the ^1^H-MRI of the studied tubes (f) and the ^2^H-MRI maps of HDO (δ = 4.7 ppm, g) and *d*_3_-thy (δ = 1.7 ppm, h). (i) Signal-to-noise (SNR) analysis of HDO and *d*_3_-thy signal intensities from ^2^H-MRI maps shown in (g) and (h) as a function of *d*_3_-thy concentration. (j) *d*_3_-thy concentration calculated from ^2^H-MRI maps shown in (h) using as an internal reference, HDO signal (17 mM, g), correlated with actual *d*_3_-thy concentrations.

The ^2^H-NMR spectrum of an aqueous solution of *d*_3_-thy revealed a clear peak resonating at 1.7 ppm, arising from the -CD_3_ group of the synthetic probe (Figure 1a, b). The 3.0 ppm upfield offset of the chemical shift of -CD_3_ deuterons relative to that of HDO (4.7 ppm) should be sufficient to spectrally resolve these ^2^H-MR signals *in vivo*, even with MR systems operating at low magnetic fields.^22^ As part of the quantification of the ^2^H-MRI experiments, the T_1_ and T_2_ relaxation times of *d*_3_-thy’s -CD_3_ were measured and found to be 283 (±50) ms and 65 (±18) ms; for an HDO resonance measured in the same agarose (4%) containing phantom, these values were 404 (±26) ms and 41 (±6) ms (Figure 1c,d).

The T_1_/T_2_ ratio of 4.3 obtained for *d*_3_-thy (Figure 1e), making this molecular probe a good candidate for ^2^H-MR studies when the data are acquired with balanced steady-state free precession (bSSFP)-based approaches,^30−32^ which were shown to allow the *in vivo* detection of sub mM levels of deuterated metabolites. To evaluate the detectability level of *d*_3_-thy when ^2^H-bSSFP-based chemical shift imaging (CSI) is used^31,32^ (Scheme S2 and Figure S1, Supporting Information), and to address the quantifiability of our ^2^H-MRI approach, a phantom consisting of low *d*_3_-thy concentrations (0.25, 0.5, and 1 mM) was studied (^1^H-MRI shown in Figure 1f). Two ^2^H-MR maps were obtained with CSI-bSSFP based on the difference in the chemical shifts of HDO (δ = 4.7 ppm, Figure 1g) and *d*_3_-thy (δ = 1.7 ppm, Figure 1h). Moreover, and significantly, the use of CSI-bSSFP to acquire the ^2^H-MR data allows to spatially map 0.25 mM of *d*_3_-thy (0.75 mM deuterons). The ability to quantify the *d*_3_-thy concentration from the ^2^H-MRI signal was then demonstrated based on the ^2^H-MRI signal of HDO (17 mM in the studied aqueous solution, relying on a 0.0156% isotopic abundance of ^2^H). Figure 1j shows the linear correlation between calculated and actual *d*_3_-thy concentration, showing high accuracy of CSI-bSSFP in calculating *d*_3_-thy concentration from ^2^H-MRI maps (for further details of the conversion of *d*_3_-thy ^2^H-MR signal amplitude to concentration, see Supporting Information). Overall, the data set presented in Figure 1 shows that the synthetic *d*_3_-thy is an optimal molecular probe for ^2^H-MRI studies, with a characteristic singlet peak of its three magnetically equivalent deuterons, which is spectrally resolved from the ^2^H-MR signal of HDO and suitable T_1_ / T_2_ ratio for enhanced detectability by CSI-bSSFP ^2^H-MRI.

### *In Vitro* Studies of the *d*_3_-thy/hTK1 Reporter System

**Figure 2:**
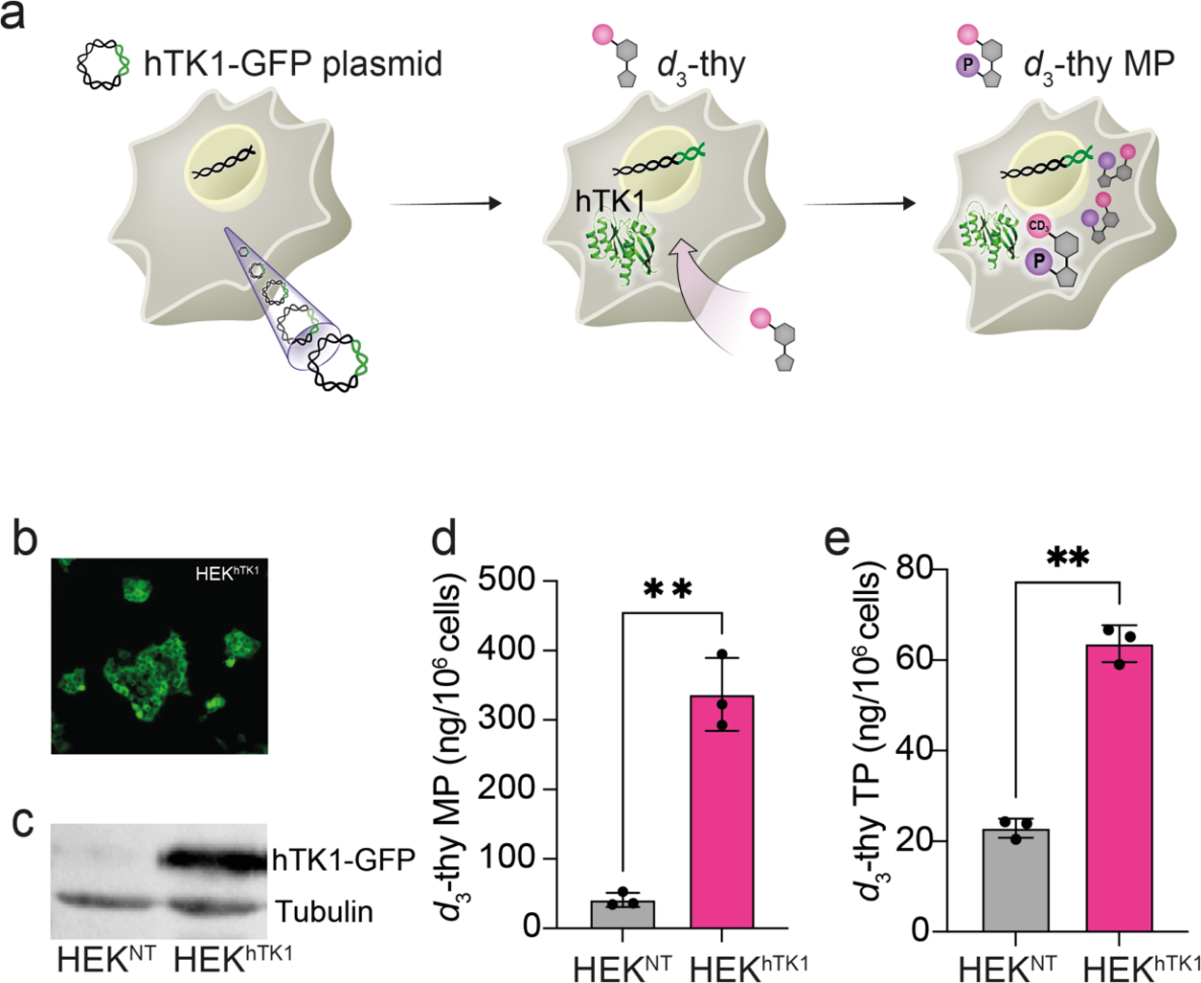
*In vitro* cellular accumulation of *d*_3_-thy in hTK1 expressing cells. (a) Schematic illustration of the proposed genetically engineered MRI reporter system based on the *d*_3_-thy/hTK1 reporter-probe/reporter-gene pair. Cellular transporters facilitate *d*_3_-thy entrance into the cells, which is then phosphorylated by hTK1 to obtain cellularly trapped *d*_3_-thy-monophosphate (*d*_3_-thy-MP). (b) Representative fluorescent image of a stable HEK-293 cell line expressing hTK1 fused to a green fluorescent protein (HEK^hTK1^). (c) Western blot analysis of lysates of nontransfected HEK-293 cells (HEK^NT^) and HEK^hTK1^ using anti-V5 antibody for hTK1-GFP expression validation and antitubulin antibody as an endogenous control. LC/MS analysis of the intracellular content of HEK^NT^ and HEK^hTK1^ cells incubated with 3 mM *d*_3_-thy solution for 4 h (*N* = 3) showing the quantities of *d*_3_-thy MP (d) and *d*_3_-thy triphosphate (*d*_3_-thy TP, e). Error bars represent the standard deviation of the mean (s.e.m). Statistics: two-tailed unpaired Student’s *t* test with ** representing a *p*-value <0.01.

To examine the hypothesis that *d*_3_-thy enters cells and accumulates upon phosphorylation by hTK1 (Figure 2a), human embryonic kidney (HEK-293) cells were stably transfected with an hTK1 transgene (fused to a green fluorescent protein, GFP) to obtain an HEK^hTK1^ stable cell line. After validation of the hTK1-GFP expression by fluorescence microscopy and Western blot examination (Figure 2b,c), and after confirmation of the tolerability of the cells to the heterologous expression and *d*_3_-thy (Figure S2), both transgenic HEK^hTK1^ cells and nontransfected (HEK^NT^) control cells were incubated with 3 mM *d*_3_-thy. Following cellular lysis, the intracellular contents of both cell lines were subjected to liquid chromatography-mass spectrometry (LC-MS) analysis to determine the accumulation of *d*_3_-thy and of its phosphorylated derivatives. As shown in Figure 2d, a significant (p-value = 0.008) 8-fold accumulation of *d*_3_-thy monophosphate (*d*_3_-thy-MP) was found in the HEK^hTK1^ cells (340 ± 50 ng/1 × 10^6^ cells) compared to control (nontransfected) HEK^NT^ cells (40 ± 10 ng/1 × 10^6^ cells), reflecting the high expression levels and high activity of the hTK1 transgene in the HEK^hTK1^ cytoplasm. The cellular *d*_3_-thy-MP was further phosphorylated in the cells by endogenous enzymes, and *d*_3_-thy -triphosphate (*d*_3_-thy-TP) was also found in significantly higher levels in HEK^hTK1^ cells compared to HEK^NT^, although at a much lower content compared to *d*_3_-thy-MP (Figure 2e, p-value = 0.0006). Note that the levels of *d*_3_-thy -diphosphate (*d*_3_-thy-DP) could not be detected in the examined cells, most probably due to the fast conversion of *d*_3_-thy-DP to *d*_3_-thy-TP. Overall, HEK^hTK1^ accumulated 23-fold more deuterated probes than the control cells.

**Figure 3:**
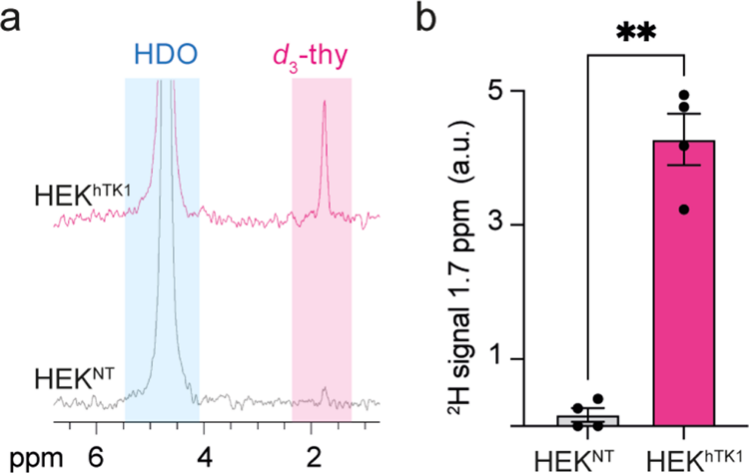
*In vitro*^2^H-NMR of *d*_3_-thy cellular accumulation in hTK1 expressing cells. (a) Representative ^2^H-NMR spectra of lysates of ten million HEK^NT^ (gray) and HEK^hTK1^ (magenta) cells, following their 3 h incubation with 5 mM *d*_3_-thy solution. (b) Quantitative analysis of the integral under the ^2^H-NMR peak at δ = 1.7 ppm assigned to the -CD_3_ of *d*_3_-thy and its phosphorylated derivatives. The integral under the 1.7 ppm ^2^H-NMR peak was normalized to that of the HDO peak (4.7 ppm). Data represent experiments performed on HEK^NT^ and HEK^hTK1^ cell lysates (*N* = 4). Error bars represent s.e.m. Statistics: two-tailed unpaired Student’s *t* test with ** representing a p-value <0.01.

The intracellular content of cells incubated with *d*_3_-thy was also examined with ^2^H NMR spectroscopy. In agreement with the LC-MS data (Figure 2), a pronounced peak was obtained at 1.7 ppm only for cells expressing hTK1 (HEK^hTK1^), with only a trace peak at this resonance obtained for the intracellular content of the control HEK^NT^ cells (Figure 3a). The absence of a deuterated signal in the ^2^H-NMR data obtained from the intracellular content of the control HEK^NT^ cells (along with the signal of endogenous HDO) suggests that there is no significant concentration of nonphosphorylated *d*_3_-thy in the cells. Thus, we concluded that the ^2^H-MR signal detected in HEK^hTK1^ is exclusively from phosphorylated *d*_3_-thy. Quantifying these results from four replicates (Figure 3b and Figure S3) showed a 25-fold higher ^2^H-NMR signal of the deuterated molecular probe in HEK^hTK1^ (Figure 3b, *p*-value = 0.0001). Note here that ^2^H-NMR counts for all *d*_3_-thy derivatives in the cells, including *d*_3_-thy, *d*_3_-thy-MP, *d*_3_-thy-DP, and *d*_3_-thy-TP; this is in contrast to MS analysis that discriminates these, based on different molecular weights (Figure 2d,e). While the different *d*_3_-thy derivatives cannot be resolved by ^2^H-NMR, one can clearly resolve the peak of these deuterated probes (1.7 ppm) from that of the HDO signal (4.7 ppm); this offers valuable potential for using the latter in quantifying the deuterated probes. To summarize these findings (Figure 2,3, and S2): *d*_3_-thy can facilitate cellular transport mechanisms to enter the cytoplasm, where it is efficiently phosphorylated by recombinant hTK1. The phosphorylated *d*_3_-thy accumulates at high levels in cells expressing the hTK1 transgene, enabling the use of the *d*_3_-thy/hTK1 pair to monitor genetically engineered reporter systems by ^2^H-MR. Thus, in contrast to other pairs of deoxyribonucleoside/deoxyribonucleoside kinase used to monitor gene expression with PET^33,34^ or CEST-MRI,^16,19,20^ the *d*_3_-thy/hTK1 pair offers the use of a native substrate/enzyme pair without compromising either the substrate structure or the enzymatic activity. Therefore, this approach reduces the need for laborious mutagenesis strategies previously applied to obtain optimal conversion of synthetic reporter probes.^20,33,35^

### *In Vivo*^2^H-MRI of *d*_3_-thy Distribution and Clearance

**Figure 4:**
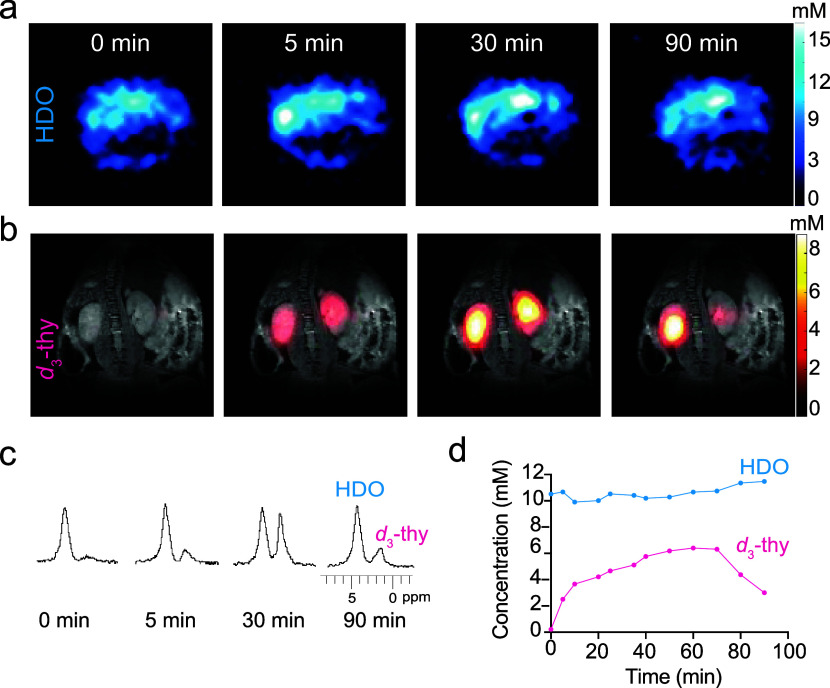
*In vivo*^2^H-MRI mapping and quantification of intravenously administered *d*_3_-thy. (a) ^2^H-MRI maps at δ = 4.7 ppm, presenting the spatial mapping of the HDO signal before (0 min) and after intravenous injection of *d*_3_-thy in PBS. (b) ^2^H-MRI maps at δ = 1.7 ppm overlaid on anatomical ^1^H-MRI of a representative mouse before (0 min) and following *d*_3_-thy administration, showing the longitudinal renal clearance of the administered probe at representative time points, 5, 30, and 90 min after the injection. (c) Nonlocalized ^2^H-NMR spectra of the studied mouse showing the characteristic signals of HDO and *d*_3_-thy over time. (d) Longitudinal data quantification of HDO (4.7 ppm) and *d*_3_-thy (1.7 ppm) signals as calculated from the ^2^H-MRI maps shown in a and b. An averaged *d*_3_-thy signal was obtained from a specific ROI covering both kidney areas and used for quantification by referring to the HDO signal detected from the same ROIs, considering a concentration of 11 mM deuterons at the natural abundance of water in animal tissue. The studied mouse was injected with 150 mg/kg body weight of *d*_3_-thy in PBS and studied on a 15.2 T MRI scanner. The data from complementary time points are shown in the Supporting Information (Figure S4).

Before examining the feasibility of mapping the genetically engineered reporter system *in vivo*, the ability to obtain quantifiable ^2^H-MRI maps of systemically administered *d*_3_-thy was evaluated through dynamic studies of the probe’s biodistribution and clearance. To this end, an anesthetized mouse was placed in the isocenter of a 15.2 T MRI scanner equipped with two 20 mm diameter surface coils designed for ^1^H-MRI (649.93 MHz) and ^2^H-MRI (99.77 MHz). Before intravenous injection of *d*_3_-thy (0 min), a clear ^2^H-MR signal of HDO was obtained (4.7 ppm, Figure 4a) with no background signal of ^2^H-containing species at 1.7 ppm (Figure 4b). Minutes after an intravenous bolus of *d*_3_-thy (150 mg/kg body weight) was injected, a clear ^2^H-NMR signal of the administered probe was observed at 1.7 ppm, as evident from the nonlocalized ^2^H-NMR spectrum of the studied mouse (Figure 4c). Spectroscopic imaging shows that this signature is localized in the kidney (Figure 4b), implying a renal clearance mechanism of *d*_3_-thy. The ^2^H-MR signal of *d*_3_-thy in the kidneys maximizes ≈30 min after the probe administration, while 90 min after *d*_3_-thy administration, both the ^2^H-NMR signal of the peak at 1.7 ppm, as well as the renal signal observed in the spatial ^2^H-MR maps, have decreased significantly. Figure 4d depicts the quantified concentration of both HDO and *d*_3_-thy, as evaluated from the ^2^H-MRI signal obtained at 4.7 and 1.7 ppm (before injection). While the HDO signal was slightly elevated following the injection of a 200 μL solution of *d*_3_-thy, probably due to the increased content of the aqueous solution going through the kidneys (Figure 4a), the concentration of the *d*_3_-thy pronouncedly changed over time. Seventy minutes after the probe administration, there was a reduction of the *d*_3_-thy level from its maximum 6 mM concentration, evidencing the renal clearance of the injected deuterated probe. These results show the feasibility of spatial mapping and longitudinally quantifying the distribution of systemically administrated *d*_3_-thy *in vivo*.

### *In Vivo* Quantitative ^2^H-MRI of hTK1 Expression

**Figure 5:**
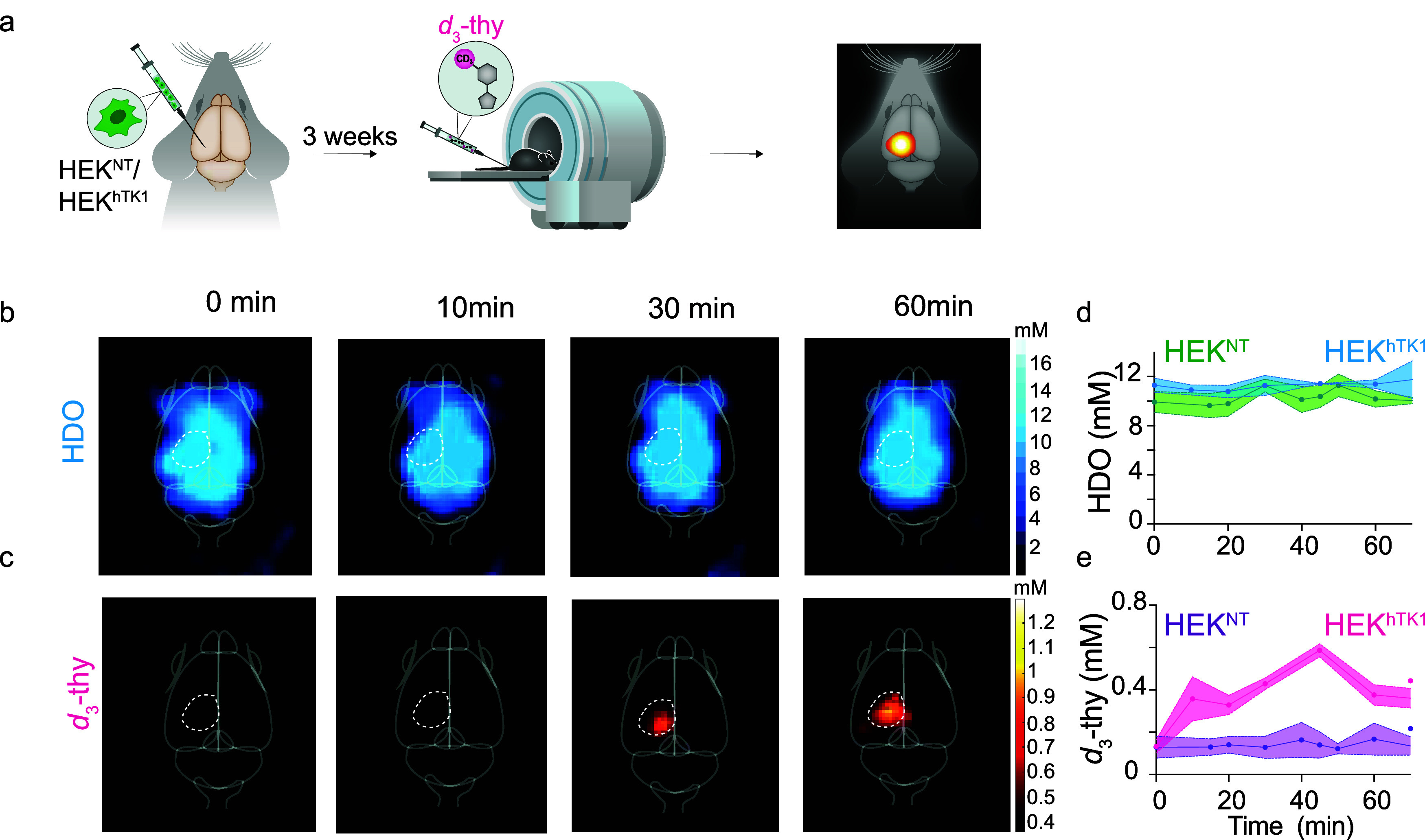
Longitudinal *in vivo*^2^H-MRI of *d*_3_-thy accumulation in hTK1 expressing tumor. (a) Schematic illustration of the *in vivo* experimental setup. Three weeks after an intracranial injection of HEK^NT^ or HEK^hTK1^ cells, the mice who had developed an HEK tumor were intravenously injected with *d*_3_-thy solution and studied by consecutive ^1^H- and ^2^H-MRI scans. (b,c) *In vivo* longitudinal ^2^H-MRI maps of a representative mouse bearing an HEK^hTK1^ tumor showing (b) ^2^H-MRI maps of HDO (δ = 4.7 ppm), and (c) ^2^H-MRI maps *d*_3_-thy (δ = 1.7 ppm). Maps are shown before (0 min) and after (10, 30, and 60 min) the probe administration. Quantification of the (d) HDO (δ = 4.7 ppm), and (e) *d*_3_-thy (δ = 1.7 ppm) ^2^H-MR signals as a function of time after the probe administration. Two groups of mice were studied, one group bearing HEK^NT^ tumors (*N* = 4) and one bearing HEK^hTK1^ tumors (*N* = 4). All mice were studied with a 15.2 T MRI scanner.

With the aforementioned results as background, we evaluated the ability of the *d*_3_-thy reporter probe to monitor the expression of the hTK1 reporter gene via the acquisition of quantitative ^2^H-MRI maps (Figure 5). To this end, either HEK293^hTK1^ or HEK293^NT^ cells (Figures 2 and 3) were intracranially injected into two groups of mice. Three weeks later, tumors reached an average size of ∼3 mm^3^, and the mice were studied with ^2^H-MRI before and after systemic delivery of the *d*_3_-thy reporter probe through the tail vein (Figure 5a). A baseline ^2^H-MRI data set was acquired before the *d*_3_-thy injection using CSI-bSSFP (same sequence as used to collect the data shown in Figure 4a,b) to obtain a ^2^H-MRI map of the endogenous HDO signal (Figure 5b, 0 min). Then, *d*_3_-thy was intravenously injected, and a series of longitudinal ^2^H-MR maps were acquired for 1 h in 8 min intervals (Figure 5b,c, and Figures S5). While the HDO ^2^H-MR signal did not change after the probe administration and throughout the experiment, a clear and gradual accumulation of the administered *d*_3_-thy was observed in the region at which the HEK293^hTK1^ were injected (Figure 5c). There was no evidence of accumulated *d*_3_-thy in other regions of the brain for any of the

studied mice (*N* = 4, Figure S6). This was by contrast to what was observed for the control group of mice inoculated with HEK^NT^ tumors, where no accumulation of *d*_3_-thy was detected in the tumor region (*N* = 4, Figure S7). Quantifying these data sets revealed an unchanged cerebral HDO concentration (quantified from the ^2^H-MR signal at δ = 4.7 ppm pre injection, Figure 5d) for both groups throughout the study. Clearly, the concentration of *d*_3_-thy (quantified from the ^2^H-MR signal at δ = 1.7 ppm, Figure 5e) was elevated after the probe administration only in HEK^hTK1^ tumors, but undetectable via the *d*_3_-thy ^2^H-MR signal in the control HEK^NT^ tumors. The maximum averaged intensity of the accumulated reporter probe *d*_3_-thy in tumors expressing the hTK1 reporter gene was reached *ca*. 40 min after the probe injection, in good agreement with other MRI reporter systems based on pairs of deoxyribonucleoside/deoxyribonucleoside kinase.^17−20^ At this time point, the *d*_3_-thy concentration was evaluated and found to be 0.6 ± 0.04 mM, corresponding to the detection of 1.8 mM deuterons (Figure 5d,e). Note that while the quantification of deuterated probe concentrations *in vivo* relies on the endogenous signal of the tissue’s HDO, further advancements are needed to improve data quality and establish a robust methodology for *in vivo*^2^H-MRI studies.

**Figure 6:**
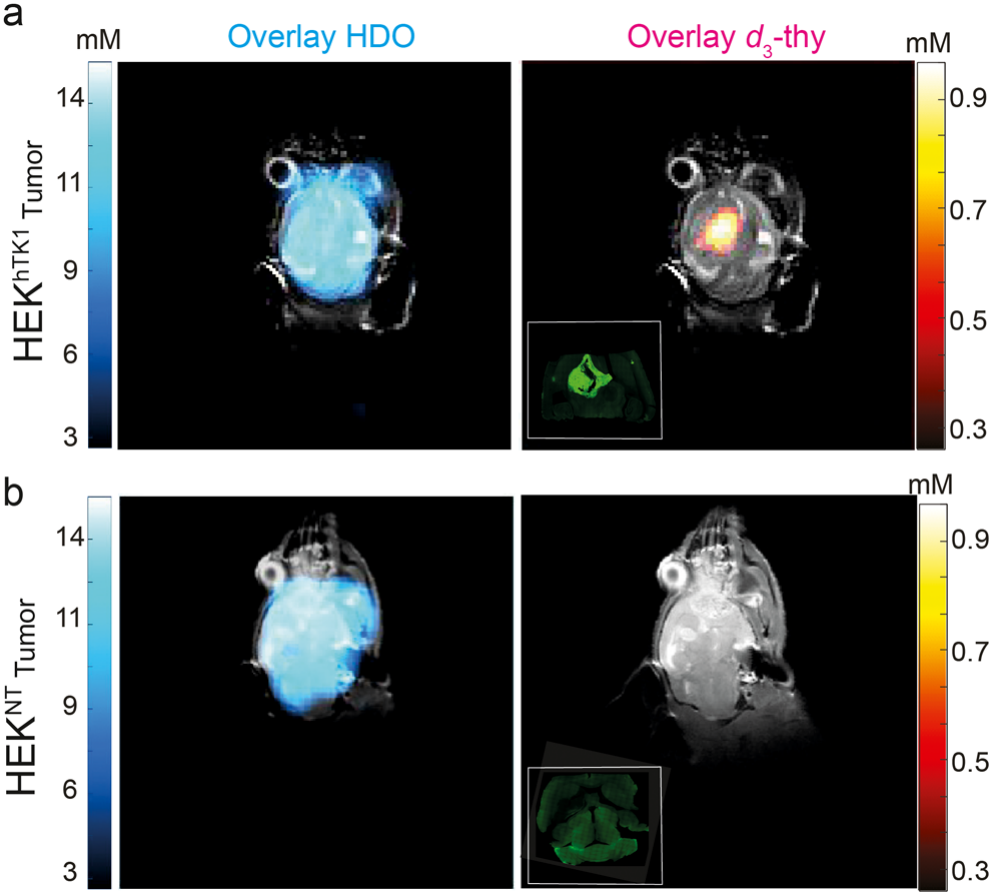
*In vivo*^2^H MRI mapping of hTK1 reporter gene expression. Representative *in vivo*^2^H-MRI maps of HDO (left) and *d*_3_-thy (right) overlaid on anatomical ^1^H-MRI of mice inoculated with HEK^hTK1^ (a), or HEK^NT^ (b) tumors. Shown are HDO (δ = 4.7 ppm) and *d*_3_-thy (δ = 1.7 ppm) maps generated by averaging four individual maps acquired at four-time points (10, 20, 30, 60) after the injection of the reporter probe. For the control mouse, a map with a lower scale bar for the *d*_3_-thy map (δ = 1.7 ppm) can be found in the Supporting Information. Fluorescent images of HEK^hTK1^ (a, inset) or HEK^NT^ (b, inset) tumor-bearing brain sections, validating the reporter gene expression solely in HEK^hTK1^ inoculated mouse.

Overlaying the summed ^2^H-MRI signal acquired over 60 min after the reporter probe injection on the ^1^H-MRI anatomical image of the same subject revealed a very good correlation between the *d*_3_-thy distribution and the tumor region, but only for the group of mice with the HEK^hTK1^ tumors (Figure 6a and Figure S6). Localized ^2^H NMR spectroscopy of a selected voxel inside the tumor tissue also showed a clear peak at δ = 1.7 ppm, while no such peak arose for a voxel taken from a normal tissue region (Figure S8). No ^2^H-MRI signal of *d*_3_-thy (δ = 1.7 ppm) could be detected in the tumors that did not express the hTK1 reporter gene (HEK^NT^, Figure 6b and Figure S7). The HDO ^2^H-MRI signal (δ = 4.7 ppm), however, was found to be equally distributed in the brain region and similar for both studied groups of mice, bearing HEK^hTK1^ (Figure 6a, left) or HEK^NT^ (Figure 6b, left) tumors. Fluorescent images of sections of excised brains of representative mice confirmed high expression levels of the hTK1 (fused to GFP) in HEK^hTK1^ tumors (Figure 6a, inset) with no fluorescent signal obtained for the control HEK^NT^ tumors (Figure 6b, inset). Overall, the results depicted in Figures 5 and 6 show that quantitative noninvasive ^2^H-MRI mapping of reporter gene expression is possible by capitalizing on the well-resolved ^2^H-MR signal of *d*_3_-thy, which is accumulated in cells expressing hTK1, making the *d*_3_-thy/hTK1 pair a favorable MRI reporter system. Nevertheless, it should be noted that the relationship between the expression levels of hTK1 and the accumulation levels of phosphorylated *d*_3_-thy should be further investigated, since cell types other than HEK-293 are expected to express lower levels of the transgene.

## Conclusions

Inspired by recent progress in ^2^H-MRI and by the desire to use structurally unmodified natural compounds as imaging reporters for natural enzymes, this study showed the design and performance of a genetically encoded MRI reporter that enabled a noninvasive, background-free and quantitative mapping of transgene expression. The study relied on the use of a synthetic *d*_3_-thy—a reporter agent that possesses multiple advantages, to map the expression of hTK1. First, *d*_3_-thy three magnetically equivalent deuterons generate a singlet ^2^H-NMR peak with preferable T_1_/T_2_ values for improved sensitivity via CSI-bSSFP sequences. Second, the 3.0 ppm offset of this peak upfield of the HDO signal provides sufficient spectral resolution to resolve the ^2^H-MR signal of the introduced *d*_3_-thy from that of the endogenous HDO signal, thus allowing signal quantification based on the known concentration of the latter in biological tissues. Third, *d*_3_-thy facilitates cellular transporters to enter cells and undergo accumulation after phosphorylation, solely in cells expressing high levels of TK1 and not in cells that do not express the transgene. This mechanism results in an enhanced contrast-to-noise ratio when mapping the conversion of *d*_3_-thy by genetically encoded hTK1. Fourth, being a natural substrate of naturally occurring enzyme, it can be used to image the expression of unmutated human protein, such as the hTK1 used here. This is a desirable quality for reporter systems when translated to clinical use, which should be intrinsically nonimmunogenic, as reported for other imaging reporters based on human proteins.^4,11,36−39^ Moreover, in contrast to synthetic analogs of thymidine commonly used to map tumor cell proliferation, which require high TK1 expression levels,^40^ the structurally unmodified thymidine (*d*_3_-thy) used here, has the potential to extend the presented approach for noninvasive quantitative monitoring of human malignancies, which overexpress hTK1,^41^ thus opening new avenues for molecular MR imaging in the future.

Finally, it is important to emphasize that despite its potential, ^2^H-MRI has certain intrinsic limitations, particularly in comparison to ^1^H-MRI and ^19^F-MRI, commonly used for molecular MR imaging. Specifically, the lower gyromagnetic ratio of ^2^H (approximately 6.5 times lower than that of ^1^H) results in two significant drawbacks: reduced spectral resolution and lower sensitivity. Consequently, the presented approach is best suited for high-field magnetic scanners, such as the 15.2 T system used in this study. As we advance, this limitation must be considered, and the applicability of this molecular imaging platform should be evaluated for MRI scanners operating at lower magnetic fields, which are more common in clinical settings.

To conclude, this work not only demonstrates a platform for quantitative mapping of reporter gene expression with noninvasive MRI but also shows the potential of ^2^H-MRI to be extended beyond its original metabolic imaging purposes to other applications for molecular imaging.

## Supporting Information Available

Experimental details, including chemical synthesis procedures, details of the NMR and MRI studies, DNA vector cloning, cell-based assays, LC-MS analysis, Western blot analysis, fluorescent microscopy setup, and *in vivo* experiments; the ^2^H-MRI data acquisition (CSI-bSSFP); data processing; and ^2^H-MRI signal quantification are also described (PDF)

## Abbreviations

MRI: magnetic resonance imaging
CEST: chemical exchange saturation transfer
DMT1: divalent metal ion transporter
HSV1-TK: herpes simplex virus type-1 thymidine kinase
*Dm*-dNK: *Drosophila melanogaster* deoxyribonucleoside kinase ^2^H-labeled thymidine (*d*_3_-thy)
HEK-293: human embryonic kidney
hTK1: human thymidine kinase-1
LC-MS: liquid chromatography-mass spectrometry
PET: positron emission tomography
GFP: green fluorescent protein
NT: non transfected
bSSFP: balanced steady-state free precession
CSI: chemical shift imaging
*d*_3_-thy-MP,: *d*_3_-thy monophosphate
*d*_3_-thy-DP: *d*_3_-thy diphosphate
*d*_3_-thy-TP: *d*_3_-thy triphosphate
PBS: phosphate buffer solution
