# Supplementary material for: A Genetically Engineered Reporter System Designed for 2H-MRI Allows Quantitative In Vivo Mapping of Transgene Expression
Source: J Am Chem Soc. 2024 Nov 11;146(46):31624–32. doi: 10.1021/jacs.4c09572 (PMC11583250; doi:10.1021/jacs.4c09572)
Supplement: Supplementary file 1 — ja4c09572_si_001.pdf [file ja4c09572_si_001.pdf]

A genetically engineered reporter system designed for ^{2}H-MRI allows quantitative *in vivo* mapping of transgene expression.

Hyla Allouche-Arnon,[a] Elton T. Montrazi,[b] Balamurugan Subramani,[a] Michal Fisler,[a] Inbal Spigel,[a] Lucio Frydman,[b] Tevie Mehlman,[c] Alexander Brandis,[c] Talia Harris,[d] and Amnon Bar-Shir[a*]

[a]Department of Molecular Chemistry and Materials Science, Weizmann Institute of Science, Rehovot, 7610001, Israel
[b]Department of Chemical and Biological Physics, Weizmann Institute of Science, Rehovot, 7610001, Israel.
[c]Department of Life Sciences Core Facilities, Weizmann Institute of Science, Rehovot, 7610001, Israel
[d]Department of Chemical Research Support, Weizmann Institute of Science, Rehovot 7610001, Israel

a. Supporting Methods

Synthesis of *d₃*-thy

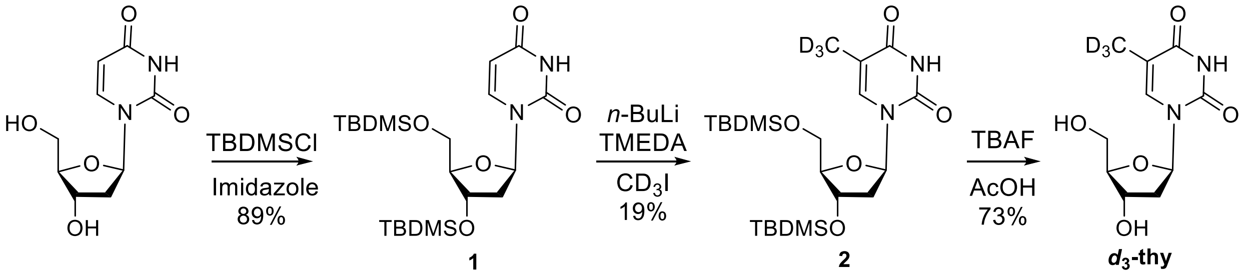

Scheme S1. Synthesis of d_3-thy

Synthesis of compound 1: Imidazole (1.49 g, 21.9 mmol) and TBDMSCl (1.98 g, 13.1 mmol) were added to the solution of 2'-Deoxyuridine (1 g, 4.3 mmol) in anhydrous DMF (15 mL) and the reaction was stirred at room temperature for 6 h. TLC monitored reaction completion and the RM was quenched with $NaHCO_3$ and extracted with ethyl acetate (3x). The combined organic layer was dried over Na_2SO_4, filtered, and concentrated under reduced pressure. The crude residue was purified by column chromatography (Ethyl acetate/Hexane, 1:5) to afford compound **1** (1.8 g, 89%) as a white solid. ^{1}H NMR (400.35 MHz, $CDCl_3$) δ 9.96 (s, 1H), 7.89 (d, J = 8.1 Hz, 1H), 6.29 (t, J = 6.1 Hz, 1H), 5.69 (d, J = 8.1 Hz, 1H), 4.40 (dt, J = 6.1, 4.1 Hz, 1H), 3.89 (dt, J = 8.4, 2.6 Hz, 2H), 3.82 – 3.69 (m, 1H), 2.32 (ddd, J = 13.2, 6.2, 4.4 Hz, 1H), 2.06 (dt, J = 12.9, 6.1 Hz, 1H), 0.91 (s, 9H), 0.88 (s, 9H), 0.09 (s, 6H), 0.07 (s, 6H). ^{13}C NMR (100.67 MHz, $CDCl_3$) δ 163.38, 150.27, 140.19, 102.18, 87.75, 85.18, 71.14, 62.40, 41.86, 25.88, 25.73, 18.35, 17.99, -4.61, -4.86, -5.50, -5.57. HRMS (ESI) calculated for $C_{21}H_{40}N_2O_5Si_2$ (M + Na): 479.2373, found: 479.2361.

Synthesis of ompound 2: Compound **1** (1 g, 21.9 mmol) was dissolved in anhydrous THF (20 ml) and cooled at -70 °C using a dry ice/acetone combination. After 10 minutes carefully the TMEDA (0.72 mL, 48.2 mmol) and n-BuLi (3.79 mL, 65.7 mmol, 1.6 M in hexane) were added dropwise and waited for 1 h at -70 °C then CD_3I (0.68 mL, 10.9 mmol) was added slowly to the reaction mixture. The resulting mixture was stirred for 1 h and slowly raised the temperature to RT. TLC monitored reaction completion, the RM was quenched with saturated NH_4Cl solution and extracted with chloroform (3x). The combined organic layer was dried over Na_2SO_4, filtered, and concentrated under reduced pressure. The crude residue was purified by column chromatography (Ethyl acetate/Hexane, 1:5) to afford compound **2** (0.2 g, 19%) as a white solid. ^{1}H NMR (400.35 MHz, $CDCl_3$) δ 9.36 – 9.06 (m, 1H), 7.48 (s, 1H), 6.35 (dd, J = 7.9, 5.9 Hz, 1H), 4.41 (dt, J = 5.8, 2.7 Hz, 1H), 3.94 (q, J = 2.5 Hz, 1H), 3.88 (dd, J = 11.4, 2.6 Hz, 1H), 3.77 (dd, J = 11.4, 2.5 Hz, 1H), 2.26 (ddd, J = 13.2, 5.9, 2.6 Hz, 1H), 2.01 (ddd, J = 13.4, 7.9, 6.0 Hz, 1H), 0.94 (s, 9H), 0.90 (s, 9H), 0.12 (s, 6H), 0.09 (d, J = 2.8 Hz, 6H). ^{13}C NMR (100.67 MHz, $CDCl_3$) δ 163.99, 150.43, 150.40, 135.51, 110.71, 87.81, 84.82, 72.24, 62.97, 41.39, 25.93, 25.76, 18.40, 18.01, -4.63, -4.83, -5.37, -5.45. ^{2}H NMR (76.76 MHz, $CHCl_3$) δ 1.85. HRMS (ESI) calculated for $C_{22}H_{39}D_3N_2O_5Si_2$ (M + Na): 496.2718, found: 496.2710.

Synthesis of *d*$_3$-thy: Acetic acid (50 µL) and TBAF (1.5 mL, 1.5 mmol, 1.0 M in toluene) were added to the solution of compound **2** (240 mg, 0.5 mmol) dissolved in anhydrous THF (5 mL). The resulting mixture was stirred at RT for 12 h. TLC monitored reaction completion and the solvent was concentrated under reduced pressure. The crude residue was purified by High-performance liquid chromatography (ACN/H_2O) and then lyophilized to afford compound ***d*$_3$-thy** (91 mg, 73%) as a white solid. ^{1}H NMR (400.35 MHz, D_2O) δ 7.56 (s, 1H), 6.20 (t, J = 6.8 Hz, 1H), 4.38 (td, J = 5.4, 4.0 Hz, 1H), 3.94 (dt, J = 5.0, 3.7 Hz, 1H), 3.81 – 3.65 (m, 2H), 2.32 – 2.24 (m, 2H). ^{13}C NMR (100.67 MHz, D_2O) δ 166.50, 151.66, 137.50, 111.27, 86.46, 84.99, 70.41, 61.14, 38.47. ^{2}H NMR (76.76 MHz, H_2O) δ 1.83. HRMS (ESI) calculated for $C_{10}H_{11}D_3N_2O_5$ (M + Na): 268.0989, found: 268.0991.

hTK1-GFP amino acid sequence:

MSCINLPTVLPGSPSKTRGQIQVILGPMFSGKSTELMRRVRRFQIAQYKCLVIKYAKDTRYSSSFC
THDRNTMEALPACLLRDVAQEALGVAVIGIDEGQFFPDIVEFCEAMANAGKTVIVAALDGTFQR
KPFGAILNLVPLAESVVKLTAVCMECFREAAYTKRLGTEKEVEVIGGADKYHSVCRLCYFKKAS
GQPAGPDNKENCPVPGKPGEAVAARKLFAPQQILQCSPANPGSIAT
MVSKGEELFTGVVPILVELDGDVNGHKFSVSGEGEGDATYGKLTLKFICTTGKLPVPWPTLVTTL
TYGVQCFSRYPDHMKQHDFFKSAMPEGYVQERTIFFKDDGNYKTRAEVKFEGDTLVNRIELKGI
DFKEDGNILGHKLEYNYNSHNVYIMADKQKNGIKVNFKIRHNIEDGSVQLADHYQQNTPIGDGP
VLLPDNHYLSTQSALSKDPNEKRDHMVLKEFVTAAGITLGMDELYK

Cloning

Human Thymidine Kinase 1 (EC 2.7.1.21, hTK1) gene was synthesized from TWIST Bioscience ltd. (San Francisco, CA, USA). The DNA fragment was subcloned into pcDNA3.1-GFP plasmid upstream of the GFP gene fragment, under the cytomegalovirus promoter to obtain pcDNA3.1-TK1-GFP expression plasmid. A V5-tag was fused following the hTK1-GFP fragments to validate hTK1-GFP protein expression by western blot analysis.

Transient cell transfection

HEK-293 cells were originally purchased from ATCC, catalog no. CRL-1573 lot no. 1353700 and provided by the tissue culture cell repository unit at the Weizmann Institute of Science.

Human embryonic kidney-293 (HEK-293) were transiently transfected using JetPEI transfection reagent (Polyplus transfection, France) with the expression plasmid pcDNA3.1-hTK1-GFP gene. Twenty-four hours post-transfection, the cells were used for substrate accumulation studies, followed by their lysis and cell content analysis by either LC-MS or ^{2}H-NMR. In addition, cell lysates were analyzed for protein expression validation.

Stable cell line generation

HEK-293 cell line was transfected with pcDNA3.1-hTK1-GFP, forty-eight hours post-transfection, GFP positive cells were sorted using FACSAria cell sorter (BD Biosciences), indicating a decent expression of TK1-GFP. Sorted cells were subsequently cultured for additional cycles of selection in the presence of geneticin (G418 Sulfate salt, Thermo Fisher Scientific, Waltham, MA, USA) at cell-type–appropriate concentration (300-700 ng/μl) in order to reach up to 95% positive cells expressing TK1-GFP protein.

TK1-GFP expression level in $HEK^{hTK1\text{-}GFP}$ was validated by fluorescent microscopy.

Western Blot Analysis

HEK-293 (HEK^{NT}) and HEK-293 expressing TK1-GFP (HEK^{hTK1}) were lysed using RIPA lysis buffer (Sigma-Aldrich Israel Ltd., Rehovot, Israel) according to the manufacturer's instruction. Protein extracts were run on sodium dodecyl sulfate polyacrylamide gel electrophoresis (SDS-PAGE) and then transferred to a nitrocellulose membrane for Western blot analysis. Western blot analyses were performed using an anti-V5 antibody (Thermo Fisher Scientific, Waltham, MA, USA) for protein expression validation and using as a loading control the cellular house-keeping protein, anti-tubulin antibodies (Santa Cruz Biotechnology Inc., Dallas, USA).

LC–MS/MS analysis of thymidine-MP and thymidine-TP

Ten million of HEK^{NT} and HEK^{hTK1}cells were incubated with a 3 mM d_3-thy for 4 h at 37°c. Cells were then washed twice with cold PBS and lysed as previously described.[2] Cells were extracted with 500uL of 60% buffered methanol (buffer: 10mM ammonium acetate and 5uM ammonium bicarbonate) in a bead beater (20 Hz, 30sec; Retsch MM400) and then in a shaker (1,500rpm, 10oC, 10min; Thermomixer C, Eppendorf), centrifuged (21,100 g, 10min). The obtained supernatant was evaporated in speedvac and then in lyophilizer. The dry residue was resuspended in 50uL of 50%-aqueous acetonitrile for LC-MS/MS analysis.

Cells content was analyzed using LC–tandem MS for phosphorylated products of d_3-thy, namely d_3-thy monophosphate (d_3-thy-MP) and d_3-thy triphosphate (d_3-thy-TP).

An Acquity I-class ultra-performance liquid chromatography system (Acquity, Waters) and triple quadrupole mass spectrometer (Xevo TQ-S, Waters) equipped with an electrospray ion source and operating in the positive ion mode was used for the analysis of deoxyribonucleosides-MP content. MassLynx and TargetLynx software (v.4.2, Waters) were used to acquire and analyze the data. The separation was achieved using BEH Z-HILIC column (2.1 x 150 mm, 1.7 um; Atlantis Premier, Waters) with 20mM ammonium carbonate buffer and acetonitrile (80:20) as mobile phase A and acetonitrile as mobile phase B in a gradient: 20%A for 0.8min, then increase to 75%A during 4.8 min, with flow rate 0.3 ml/min. d_3-thy-MP and d_3-thy-TP were detected using multiple reaction monitoring with the following

parameters: 325.9 > 80.9 and 322.9 > 80.9 m/z (collision energy, CE 16 eV) for d_3-thy-MP and regular thy-MP, respectively, and 486.0 > 81.0 (CE 12 eV), 486.0 > 210.0 (CE 6 eV) m/z for d_3-thy-TP, and 483.0 > 81.0 (CE 12 eV), 483.0 > 207.0 (CE 6 eV) m/z for regular thy-TP. The absolute concentrations of d_3-thy-MP and d_3-thy-TP were determined using relevant standard curves for regular thy-MP and thy-TP, with adenine-15N5 5′-MP and adenine-13C10 5'-TP as the internal standards (Sigma Aldrich, catalog no. 662658 and 710695), having 353.1 > 97.0 (CE 28 eV), 353.1 > 141.1 (CE 20 eV) m/z and 518.1 > 141.1 (CE 35 eV), 518.1 > 420.1 (CE 18 eV) m/z.

^{2}H-NMR of *d*$_3$-thy solution

^{2}H-NMR spectra of 5 mM d_3-thy aqueous solution was measured on a Bruker 400 MHz spectrometer, resolving 2 singlet peaks of HDO (δ = 4.7 ppm) and d_3-thy (δ = 1.7 ppm).
T_1 and T_2 relaxation times of HDO and d_3-thy signals detected from a 5 mM of d_3-thy solution dissolved in 4% agarose, were measured by ^{2}H NMR on a Bruker 600 MHz spectrometer.

***In vitro* ^{2}H-NMR of *d*$_3$-thy cellular accumulation**

Ten million HEK^{NT} (N=4) or HEK$^{hTK1\text{-}GFP}$ (N=4) stable cell lines were incubated in cell-culture media containing a 5 mM of d_3-thy solution. Following 3 h of incubation (37 °C, 5% CO_2), the cells were lysed using BugBuster Protein Extraction Reagent (Merck Israel, Cat. Num. 70584) according to the manufacturer's instructions. The lysed content in the aqueous phase was transferred to a 5 mm NMR tube. A ^{2}H-NMR spectra of the extracts were acquired on a Bruker 400 MHz spectrometer. The signal integrals of d_3-thy peak at δ= 1.7 ppm was determined and normalized to the HDO peak at δ = 4.7 ppm as a reference.

Animal care

All experiments involving animals were performed following the described experimental protocol approved by the Weizmann Institute of Sciences Animal Care and Use Committee (IACUC approval number 04660522-2). All animals were kept in a daily controlled room at the Weizmann Institute of Sciences animal facility with a surrounding relative humidity level of 50 ± 10% and a temperature of 22 ± 1 °C, with a 12/12 cycle of dark and light phases.

HEK-293 tumor inoculations

For the intracranial tumor xenograft model, either stable HEK^{NT} or HEK$^{hTK1\text{-}GFP}$ cells lines were intracranially inoculated (2×10^5 cells per 2 µl of serum-free medium) into the left striatum (coordinates 1 mm posterior to Bregman, 2.0 mm lateral to the midline and 3.0 mm ventral to the surface of the skull) of 8-week-old female immunodeficient Hsd:Athymic Nude-Foxn1nu mice (Envigo), which generated intracranial tumors in left hemisphere.

^{2}H-MRI of *d*$_3$-thy solutions

A phantom comprising tubes of d_3-thy solutions (0.25, 0.5, and 1 mM) and PBS, dissolved in 4% agarose, was located in a horizontal 15.2 T MRI scanner (Biospec, Bruker) running ParaVision v.6.0.1 software. ^{1}H and ^{2}H-MRI of the phantom were acquired using RARE and CSI-SSFP sequences, respectively.

^1H and ^2H MRI and MRS

All $^2H/^1H$ measurements were performed on a 15.2 T Bruker scanner running ParaVision v.6.0.1 software, using 20 mm diameter surface coils tuned to 649.93 (^1H) and 99.77 MHz (^2H).

^1H MRI

Phantom imaging: ^1H axial images of the d_3-thy tubes were acquired using a RARE pulse sequence (TR/TE = 6,000/20 ms, RARE factor 8, 10 mm slice thickness, FOV = 20 × 20 mm^2, matrix size 256 × 256 encoding matrix NA = 2).

Mouse brain imaging: ^1H coronal anatomical images of the mouse brain were acquired for localization using a RARE pulse sequence (TR/TE = 6,000/20 ms, RARE factor 8, 1 mm slice thickness, FOV = 40 × 4 0 mm^2, matrix size 256 × 256 encoding matrix NA = 2).

^2H MRI (CSI-bSSFP)

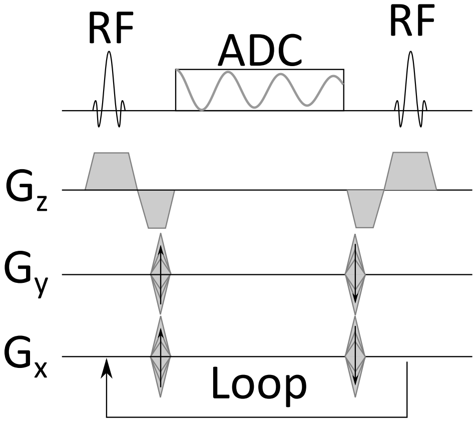

Scheme S2. CSI-bSSFP sequence. The CSI-bSSFP pulse sequence is based on a regular balanced Steady-State Free Precession (bSSFP) sequence added with phase gradients in two directions (G_y and G_x) for the spatial encoding, which is conventionally applied in the CSI pulse sequence.

^2H MRI data of d_3-thy phantom and mouse brain were acquired using the CSI-bSSFP sequence (ref-sequence available at https://www.weizmann.ac.il/chembiophys/Frydman_group/software). Supporting Scheme 2. illustrates the main component of the CSI-SSFP sequence, which is a conventional balanced SSFP sequence with phase gradients in two (or three) directions for the spatial encoding similar to the one applied at CSI sequence. The acquisition parameters were used as the following: 1.7 ppm carrier frequency, TR = 6.5 ms, flip angle = 60°, 32 × 32 matrices, in-plane FOV = 20 × 20 mm^2, 10 mm slice (d_3-thy solutions) or FOV = 40 × 40 mm^2 (mouse kidneys and brain), 20 mm slice. For all ^2H - CSI-SSFP experiments 33-points were sampled in the gradient-free FIDs at 3.2 kHz (out of which the 4 initial points had to be discarded as they were corrupted by the digital filtering). Weighted signal averaging of the phase-encoding (PE) domains was applied using 16 repetitions and weighting coefficient NA = 4, signal averaging was thus ~ 14 min for each time point. Spatial domains for CSI-SSFP experiments were reconstructed by 2D Fourier transform after zero-filling to 64 × 64 points. Images arising from each CSI-SSFP FID point were processed using the Water/Fat Separation Algorithm[3] from the FAT-WATER TOOLBOX (MathWorks),[3] which isolated the images of the individual sites using a priori known chemical shift positions of $\Delta\omega$ = 4.7, and 1.7 ppm for HDO and d_3-thy, respectively. To translate the intensities into metabolic concentrations, ^2H's natural abundance (~ 11 mM concentration before injection in biological tissue), together with SSFP's signal attenuations as expected from the scanning parameters and the T_1/T_2 for each species, were used as previously described.[4]

^{2}H MRS

^{2}H Spectroscopic data was acquired from isotropic 3 mm^3 voxel in and out of the brain tumor region of HEK$^{hTK1-GFP}$ tumor-bearing mouse, using Image Selected In vivo spectroscopy (ISIS) sequence, acquiring 1024 points with TR=1200 mesc, averaging of 400 scans, for a total scan time of 1hour.

The image processing FIJI software [1] was used to overlay the ^{2}H MRI maps on an anatomical ^{1}H-magnetic resonance image for spatial display of the detected ^{2}H signal at δ = 4.7, and 1.7 ppm for HDO and *d*$_3$-thy signals, respectively.

***In vivo* ^{1}H/ ^{2}H MRI**

All in vivo experiments were performed on anesthetized mice (1.5% isoflurane) that were placed in a horizontal 15.2 T preclinical MRI scanner (Biospec, Bruker). Mice were monitored for their breathing throughout the MRI session, using a dedicated respiratory monitor setup. Three weeks after HEK^{NT} or HEK$^{hTK1-GFP}$ cells transplantation, ^{2}H MRI datasets were acquired from kidneys mouse (N=1), before and during 90 min following the intravenous injection of a 100 mM *d*$_3$-thy solution (200 µl in saline, 150 mg kg^{-1}). Longitudinal monitoring of *d*$_3$-thy renal clearance was observed by acquiring sequential ^{2}H-MRI data (CSI-bSSFP as described above) every 10 min following probe administration. For tumor-bearing brain imaging experiments. Three weeks after either, HEK^{NT} (N=4) or HEK$^{hTK1-GFP}$ (N=4) cells transplantation, ^{2}H MRI datasets were acquired before and following the intravenous injection of a 100 mM *d*$_3$-thy solution (200 µl in saline, 150 mg kg^{-1}). High-resolution ^{1}H MRI images (details above) were acquired using the same FOV of ^{2}H MRI images, allowing the spatial localization of the ^{2}H signal in the kidneys or the brain tumor region.

Quantification of *d*$_3$-thy *in vitro* and *in vivo* from ^{2}H CSI-bSSFP data

The quantification was performed with a custom-designed MATLAB script (MathWorks).

The amplitude values in the HDO (A_{HDO}) and *d*$_3$-thy ($A_{d3-\mathrm{thy}}$) ^{2}H-MRI maps are proportional to the bSSFP signals of HDO and *d*$_3$-thy and their concentrations μ:

$$A_{d3-\mathrm{thy}} = C \cdot S_{SSFP}^{d3-\mathrm{thy}} \cdot \mu_{d3-\mathrm{thy}} \quad (1)$$

$$A_{\mathrm{HDO}} = C \cdot S_{SSFP}^{\mathrm{HDO}} \cdot \mu_{\mathrm{HDO}} \quad (2)$$

C is a constant of proportionality that depends on the spectrometer.

The bSSFP signal depends on T_1, T_2, T_R, and flip angle α according to the following equation:

$$S_{SSFP} = \frac{\sqrt{E_2} \cdot (1 - E_1) \cdot \sin(\alpha)}{1 - (E_1 - E_2) \cdot \cos(\alpha) - E_1 \cdot E_2} \quad (3)$$

where $E_1 = \exp(-T_R/T_1)$ and $E_2 = \exp(-T_R/T_2)$.

In order to determine the C constant, we first calculated the S_{SSFP}^{HDO}, from a selected a region of interest (ROI). Then, the averaged amplitude was divided by HDO concentration 17 mM (*in vitro*) or 11 mM (*in vivo*). Using the appropriate T_1, T_2, T_R, and flip angle α parameters, S_{SSFP}^{HDO} was calculated from the selected ROI using Eq. 3. and C was determined according to Eq. 4

$$C \cdot S_{SSFP}^{\mathrm{HDO}} = \frac{\langle A_{\mathrm{HDO}} \rangle_{\mathrm{ROI}}}{\mu_{\mathrm{HDO}}} \quad (4)$$

The water concentration in aqueous solution, *in vitro,* was estimated to be 17 mM, relying on the natural abundance of deuterium of 0.0156% and 55 M of water molecules. However, the HDO concentration *in vivo* was estimated to be 11 mM, assuming 65% presence of water in biological tissue.
Consequently, when C was calculated for a certain ROI containing HDO signal, the d_3-thy amplitude map was converted to concentration using Eq. 1.

Fluorescence microscopy of brain sections

Following *in vivo* MRI studies, mice were anesthetized (75 mg kg^{-1} ketamine, 1 mg kg^{-1} dexmedetomidine in 0.9% saline) and perfused transcardially with 10 mM PBS. Mice brains were then carefully removed and fixed with a 2.5% paraformaldehyde (Santa Cruz Biotechnology, catalog no. sc-281692) solution (wt/vol) for 24 h, followed by their submersion in a 30% (wt./vol.) sucrose solution (in PBS) for 24–48 h for cryogenic preservation. Brains were washed with PBS to remove any excess sucrose solution and immediately frozen on dry ice and preserved at −80 °C. Excised brains were cut into 40-µm-thick slices using a dedicated cryotome. The appropriate slices were mounted on microscope slides and imaged using an inverted Leica DMI8 wide-field fluorescent microscope. Consecutive tile stitching with a 10% spatial overlap, according to Leica image analysis software, was generated to obtain complete coverage of the brain slices' FOV. All fluorescent images were analyzed and processed using FIJI software [2] TK1-GFP expression levels in the brain tumors of the examined mice were imaged using $\lambda_{\mathrm{excitation}} = 400$ nm, $\lambda_{\mathrm{emission}} = 510$ nm for GFP detection.

Data analysis and graphic presentation

All the graphical data presented in the paper were analyzed and generated using Prism v.7 software (Graphpad).

Statistical analysis

All numerical results are presented in the form of mean ± s.e.m. and unpaired Student's *t*-test was used for statistical comparison and significance evaluations (as noted in the figure captions). All statistical evaluations were performed using two-tailed analysis unless noted differently in the figure caption.

b. Supporting Figures

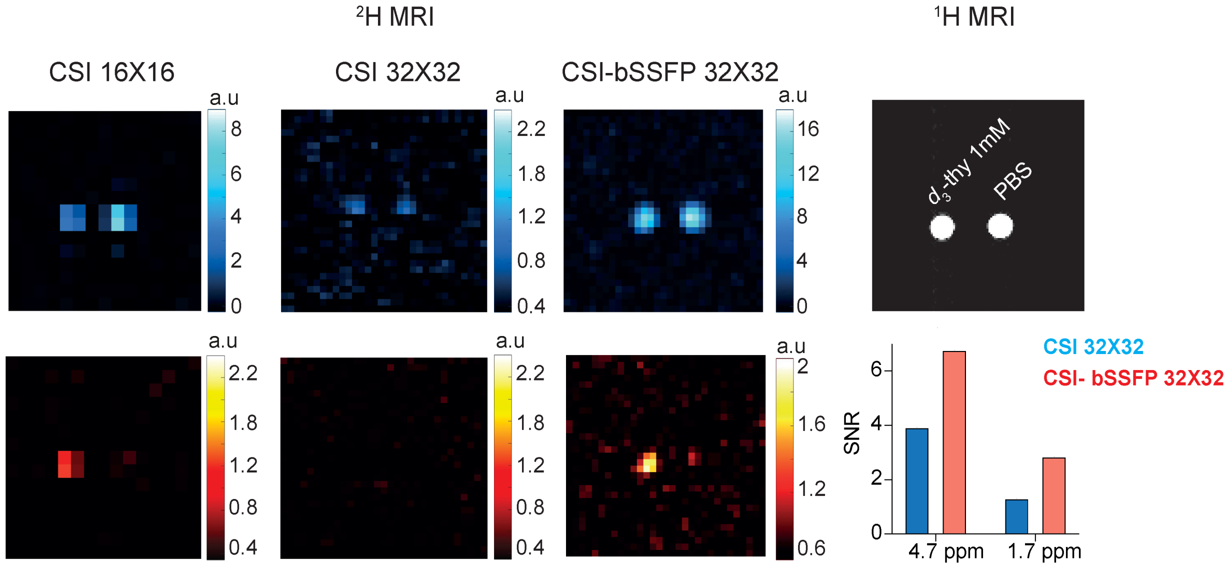

Figure S1. SNR of ^{2}H-MRI data (CSI vs. CSI-bSSFP). Illustration of the significant SNR improvements of ^{2}H CSI-bSSFP versus traditional ^{2}H CSI on the methyl peak of d_3-thy (1.7 ppm). Shown is experimental data obtained at two different spatial resolutions (as noted in the Figure) of phantom composed of two tubes of 4% (w/v) agarose solution—one containing 1 mM of d_3-thy (left) and one without (right, reflecting solely the naturally abundant HDO). The ^{2}H-maps of HDO (top row, ^{2}H-MR signal at 4.7 ppm, colored blue) and of d_3-thy (bottom row, ^{2}H-MR signal at 1.7 ppm, colored red hot). The ^{1}H-MR image at the top-right shows a similar water signal of the two examined samples. The plot at the right bottom represents the SNR calculated from ^{2}H-CSI or ^{2}H CSI-bSSFP with the same resolution (matrix of 32 × 32 for FOV of 40 × 40). ^{2}H CSI-bSSFP generated a 1.8 higher ^{2}H-d_3-thy signal than conventional ^{2}H-CSI when the same flip angle (60°) and scan time were applied. This reflects the T_1/T_2 ratio characterizing ^{2}H in the methyl group of this probe. A clear localization of d_3-thy at the employed resolution (1.25 × 1.25 mm²) could only be observed using CSI-bSSFP. Importantly, with conventional ^{2}H-CSI, the location of d_3-thy could be detected only when the resolution was significantly reduced (16 × 16 matrix size with the same FOV) to 2 × 2 mm². By contrast, the enhancement in HDO is more modest –as also expected when applying CSI-bSSFP to ^{2}H sites in this T_2<<T_1 situation.

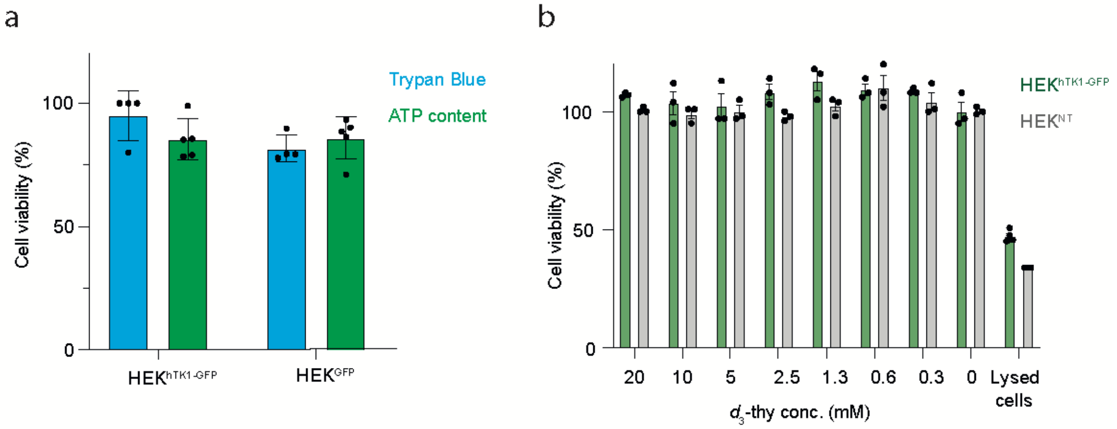

Figure S2. Cell viability assays. (a) Cellular viability of HEK$^{TK1\text{-}GFP}$ and HEK^{GFP} cells stably expressing the transgenes TK1-GFP or GFP. The viability of the cells was measured using two independent assays: Trypan Blue (blue) for the evaluation of live dead cell ratio, and cell-titer glo assay to measure ATP in viable cells (green). Data was obtained from cultured cells during 48 hours without the replacement of their culture medium, indicating viable cells in both transgenes cell lines. For the Trypan Blue assay, cells were diluted in Trypan Blue solution (1:1 v/v) followed by cell counting of viable or dead cells. (b) the effect of d_3-thy on the cell viability, measured Cell Titer Blue assay. Data was obtained from cells incubated with 0.3-20 mM of d_3-thy for 3 hours. 50% DMSO was used as a positive control to represent a cell toxicity scenario. (n=3 biologically independent experiments). Data are presented as mean values ± s.e.m.

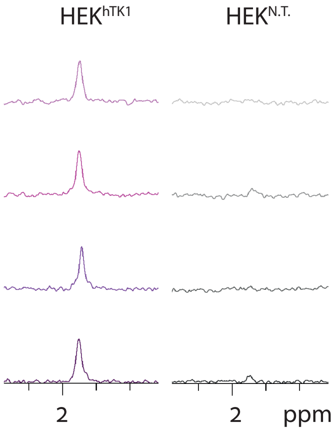

Figure S3. In vitro d_3-thy cellular accumulation in hTK1 expressing cells.
Four independent replicates of accumulation assay of HEK^{hTK} and HEK^{NT} transgene cells with d_3-thy. Data shown are the ^{2}H-NMR spectra of cells lysates from HEK^{hTK1} (N=4) and HEK^{NT} (N=4) stable cell line following their incubation with 5 mM d_3-thy solution for 3 hours, depicting singlet peak at δ=1.7 ppm pointing on high d_3-thy signal solely in HEK^{hTK1} cells compared to HEK^{NT}.

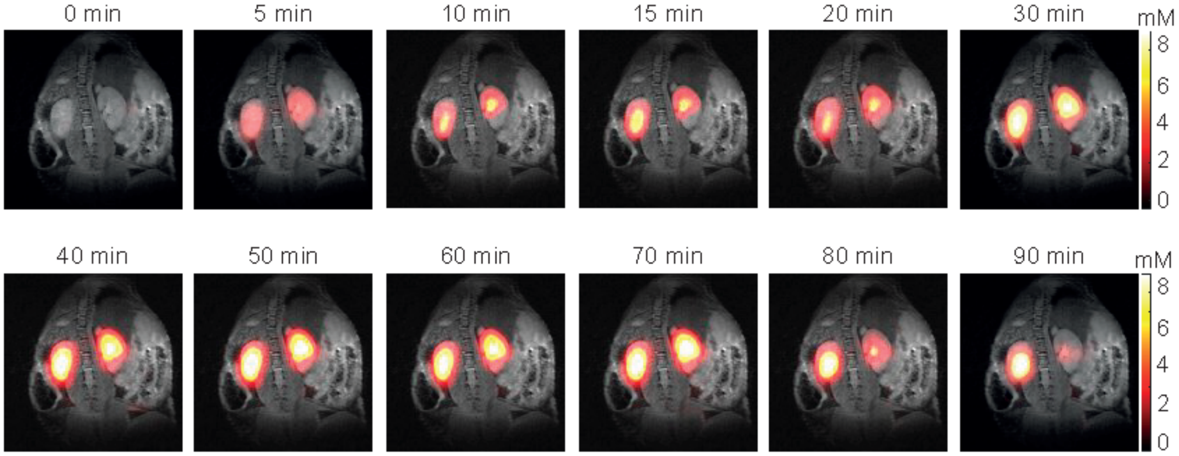

Figure S4. Longitudinal *in vivo* d_3-thy renal clearance.
Sequential ^{2}H-MRI maps at δ=1.7 ppm, overlaid on anatomical ^{1}H-MRI before (0 min) and at all acquired time points following d_3-thy administration (5,10, 15, 20, 30 40, 50, 60, 70, 80, and 90 min). The longitudinal quantification of d_3-thy maps demonstrated the renal clearnace mechanism of d_3-thy over time.

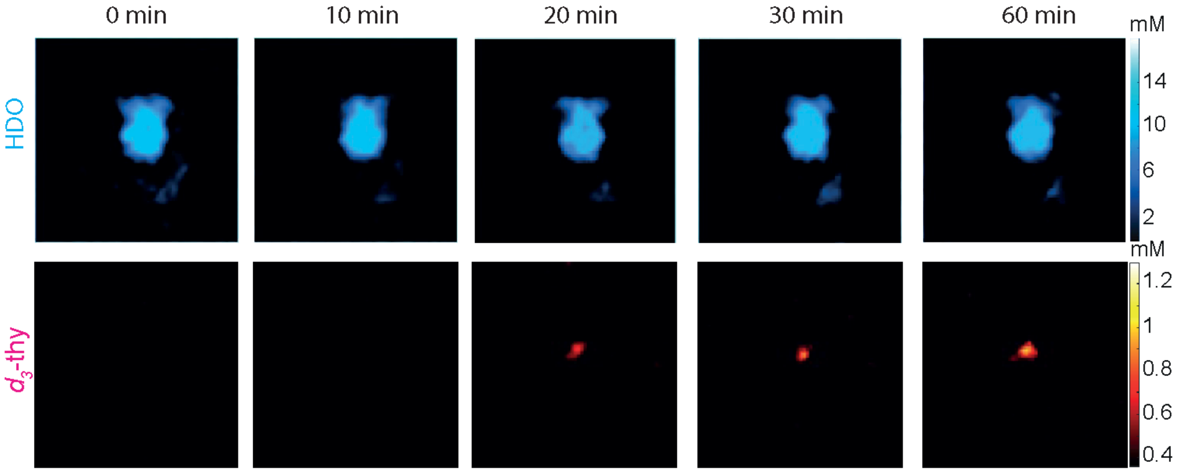

Figure S5. Longitudinal *in vivo* ^{2}H-MRI of d_3-thy accumulation in hTK1 expressing tumor.
Sequential ^{2}H-MRI maps of HDO (δ=4.7 ppm, upper), and ^{2}H-MRI maps of d_3-thy (δ=1.7 ppm, lower) before (0 min) and following d_3-thy administration (5,10, 20, 30, and 60 min). Longitudinal ^{2}H-MRI data shown points on the increasing d_3-thy accumulation in HEK^{hTK1} tumor- bearing mouse, while HDO signal stays stable over time and is equivalently present in all animal brain.

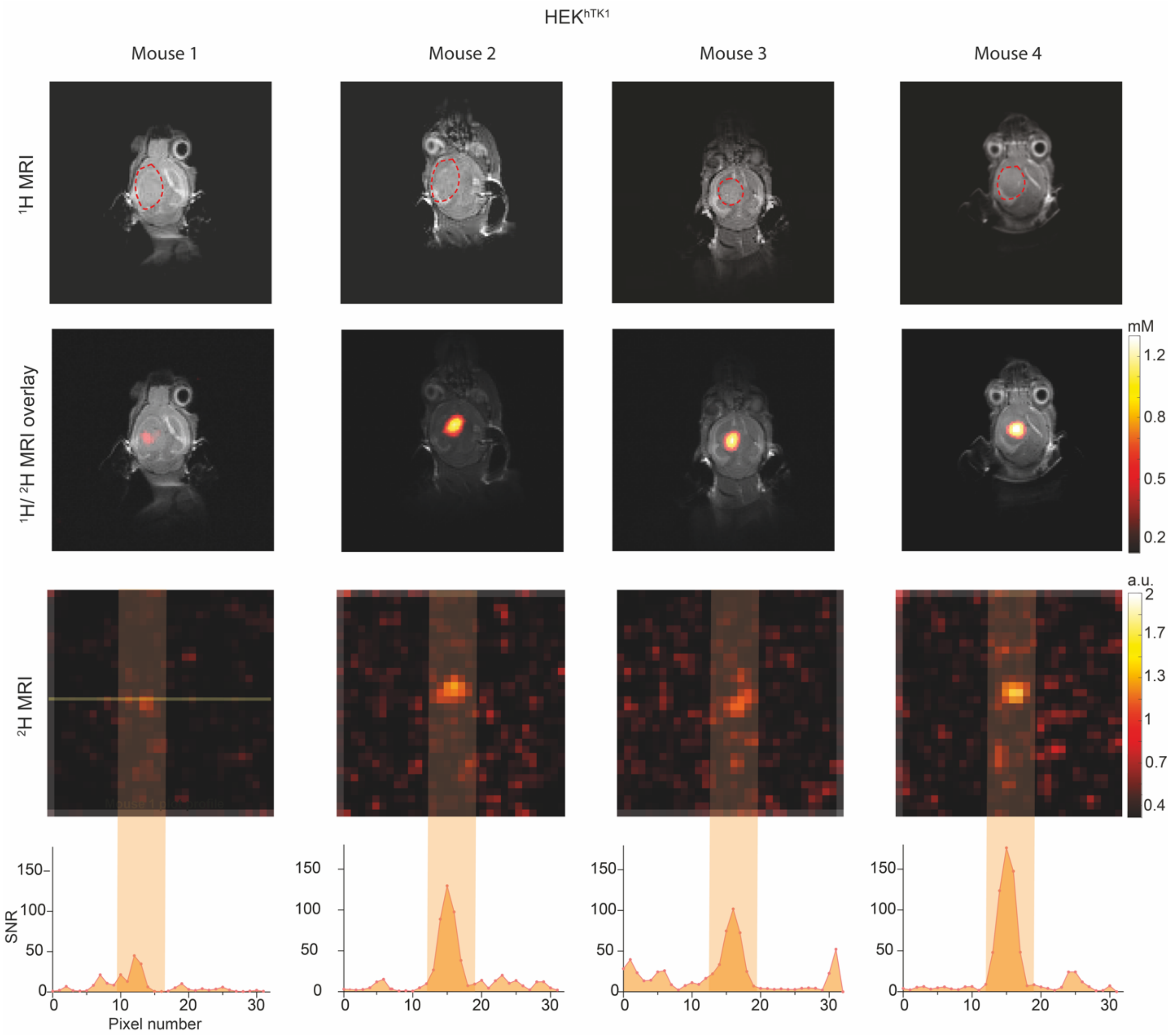

Figure S6. *In vivo* ^{2}H MRI mapping of hTK1 reporter gene expression.
Top row: *In vivo* ^{1}H-MR images of the studied mice, showing the tumor region outlined in red.
Middle row*:* *In vivo* ^{2}H-MRI maps of averaged d_3-thy signal (δ=1.7 ppm) overlaid on anatomical ^{1}H-MRI of four mice inoculated with HEK^{hTK1} tumors (N=4). In all cases, sequential d_3-thy maps detected at δ=1.7 ppm following the probe administration were averaged to a single d_3-thy ^{2}H-MRI map that was overlaid on^{1}H-MRI displaying the spatial mapping of hTK1 gene expression. (Mouse 1- 7 time points averaged map, mouse 2 - 9 time points averaged map, mouse 3 and mouse 4- 5 time points averaged map).
Bottom row: The profile of the signal-to-noise ratio (SNR) across all ^{2}H-MRI maps of the *in vivo* data. Specifically, it shows the SNR profile of the ^{2}H-MR signal at 1.7 ppm, representing the d_3-thy distribution for each scanned mouse. This profile provides a detailed, unbiased analysis of the regions where the ^{2}H-MR signal is significantly above the noise level, corresponding to the tumor region and d_3-thy localization.

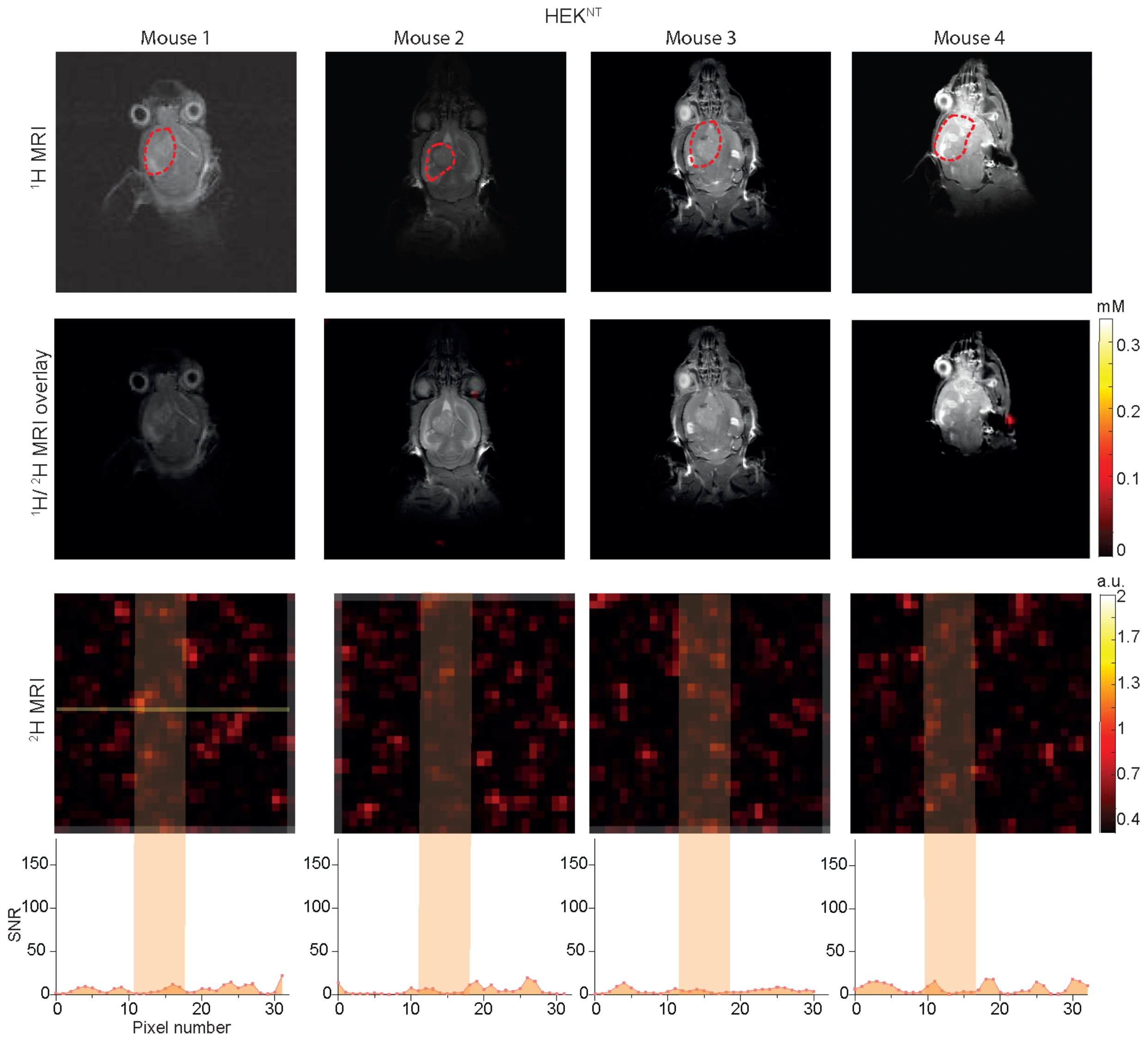

Figure S7. ***In vivo*** ^{2}**H MRI of HEK**^{NT} **tumor bearing mice.**

Top row: *In vivo* ^{1}H-MR images of the studied mice, showing the tumor region outlined in red.

Middle row*: In vivo* ^{2}H-MRI maps of averaged d_3-thy signal (δ=1.7 ppm) overlaid on anatomical ^{1}H-MRI of four mice inoculated with HEK^{NT} tumors (N=4). In all cases, sequential d_3-thy maps detected at δ=1.7 ppm following the probe administration were averaged to a single d_3-thy ^{2}H-MRI map that was overlaid on^{1}H-MRI displaying the spatial mapping of hTK1 gene expression. (Mouse 1- 7 time points averaged map, mouse 2 - 9 time points averaged map, mouse 3 and mouse 4- 5 time points averaged map).

Bottom row: The profile of the signal-to-noise ratio (SNR) across all ^{2}H-MRI maps of the *in vivo* data. Specifically, it shows the SNR profile of the ^{2}H-MR signal at 1.7 ppm, representing the d_3-thy distribution for each scanned mouse. This profile provides a detailed, unbiased analysis of the ^{2}H-MR signal across the FOV.

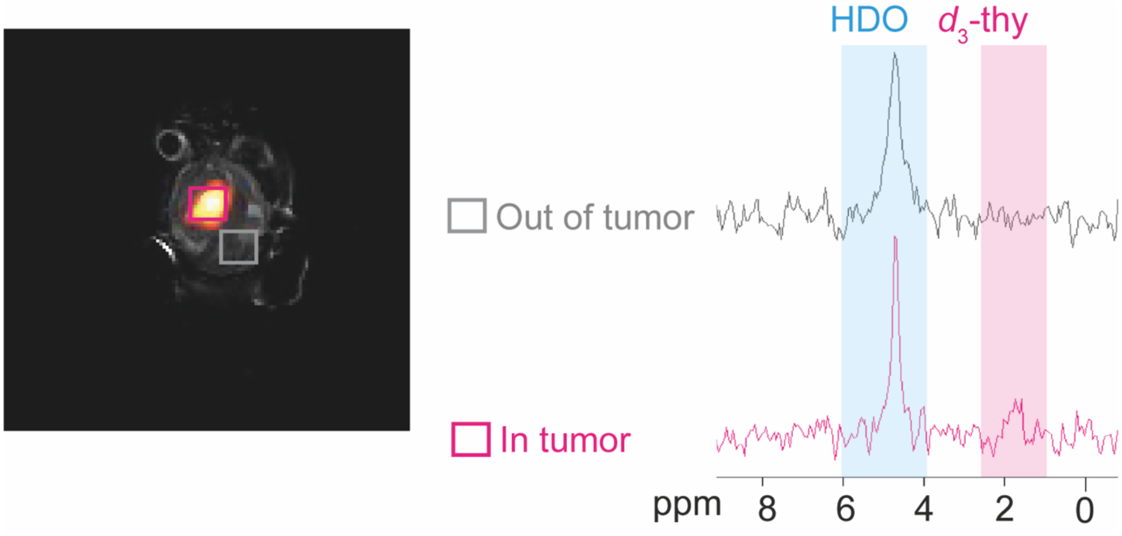

Figure S8. Localized ^{2}H spectroscopy of HEK^{hTK1} brain tumor.
^{2}H-NMR spectra acquired from 3 mm^3 voxels located in (magenta) and out (gray) of HEK^{hTK1} tumor region, showing the presence of HDO and d_3-thy peak depicted at δ=4.7 ppm and δ=1.7 ppm in the tumor region compared to only HDO signal out of the tumor region.

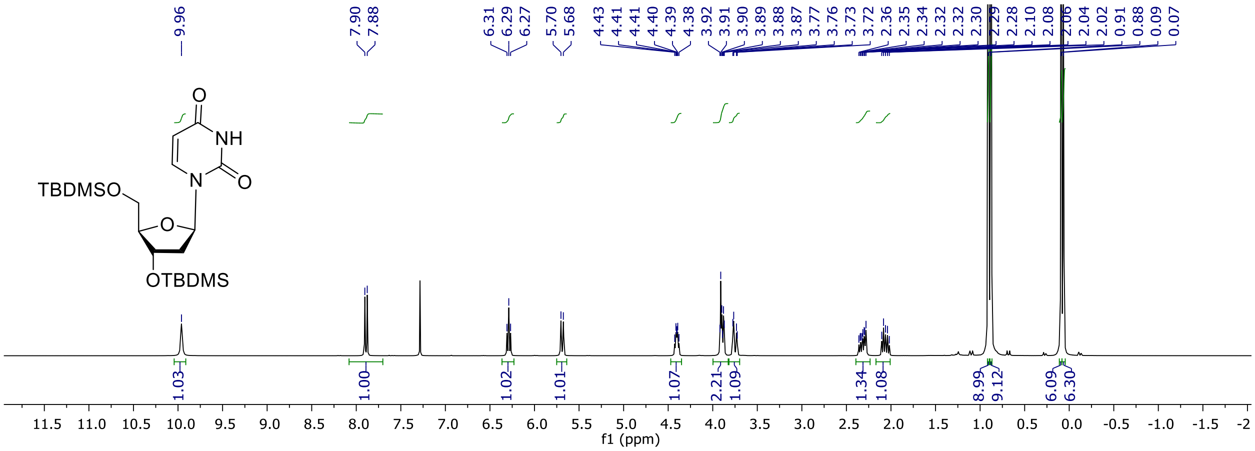

Figure S9: ^{1}H NMR (400.35 MHz, $CDCl_3$, 25 °C) spectrum of compound **1**.

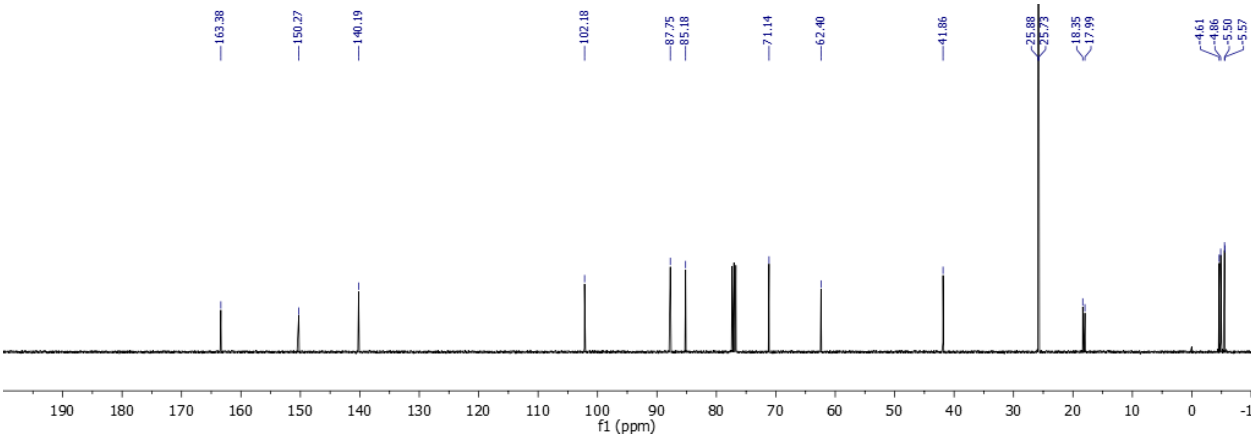

Figure S10: ^{13}C[2] NMR (100.67 MHz, $CDCl_3$, 25 °C) spectrum of compound **1**.

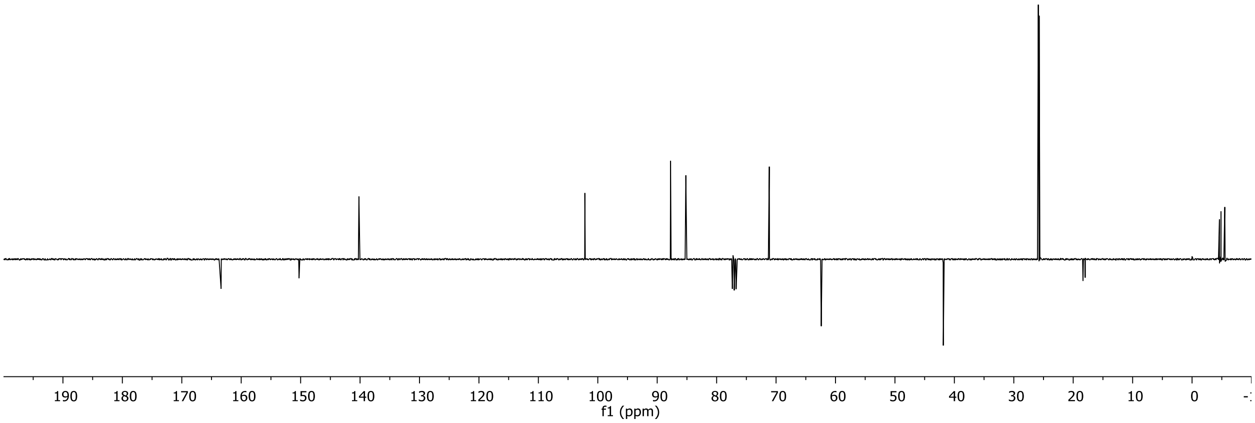

Figure S11: DEPTQ[2] NMR (100.67 MHz, $CDCl_3$, 25 °C) spectrum of compound **1**.

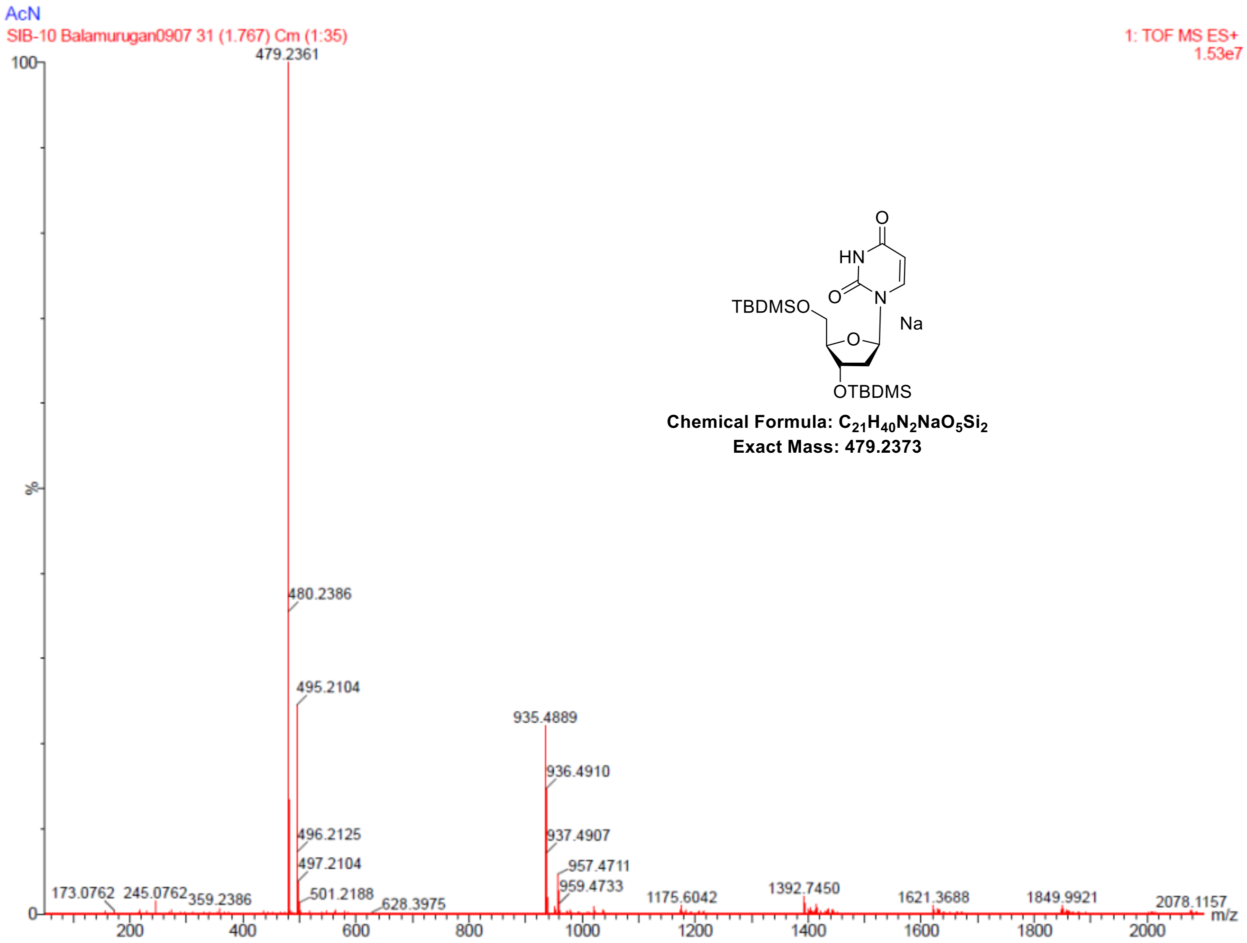

Figure S12: HRMS (ESI) spectrum of compound **1**.

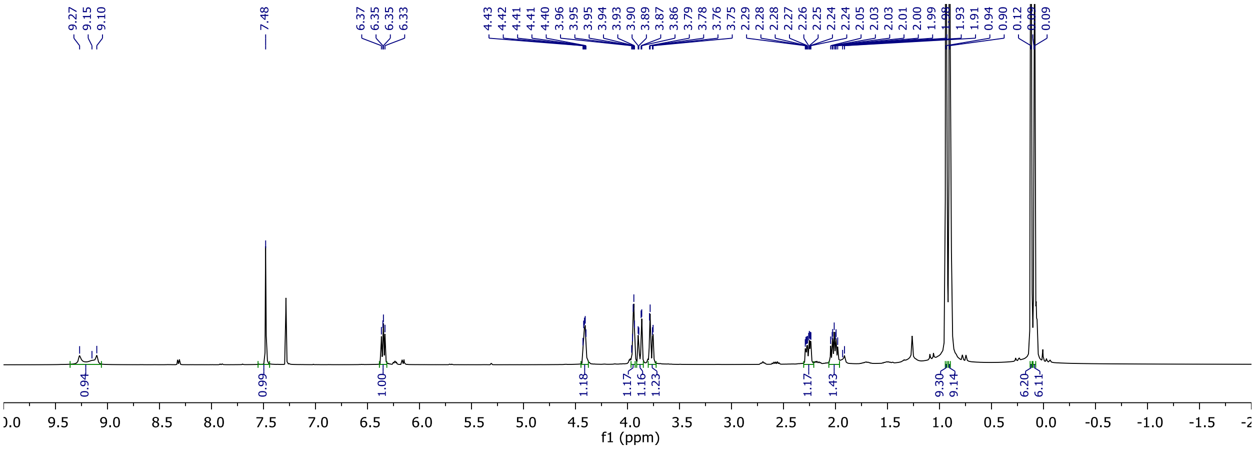

Figure S13: ^{1}H NMR (400.35 MHz, $CDCl_3$, 25 °C) spectrum of compound **2**.

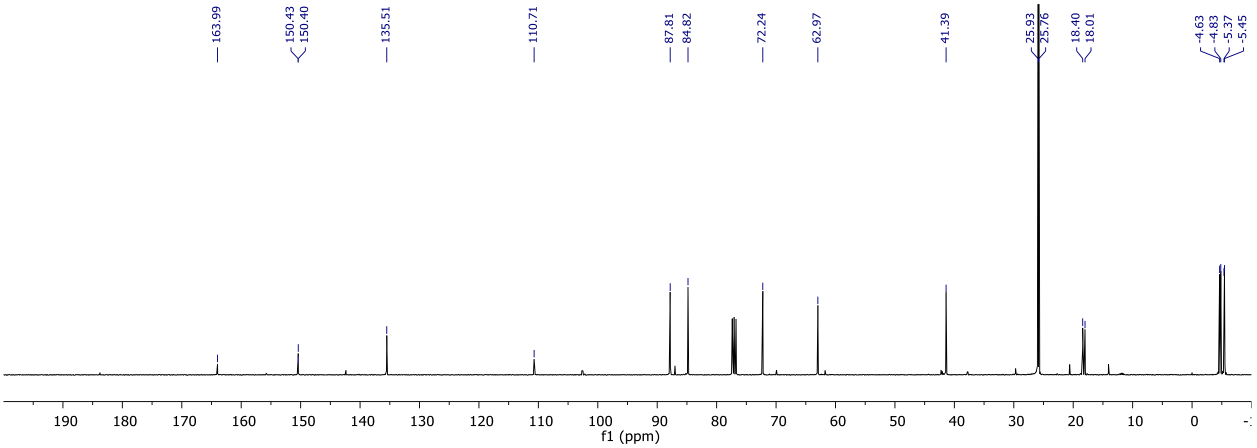

Figure S14: $^{13}C^{2}$ NMR (100.67 MHz, $CDCl_3$, 25 °C) spectrum of compound **2**.

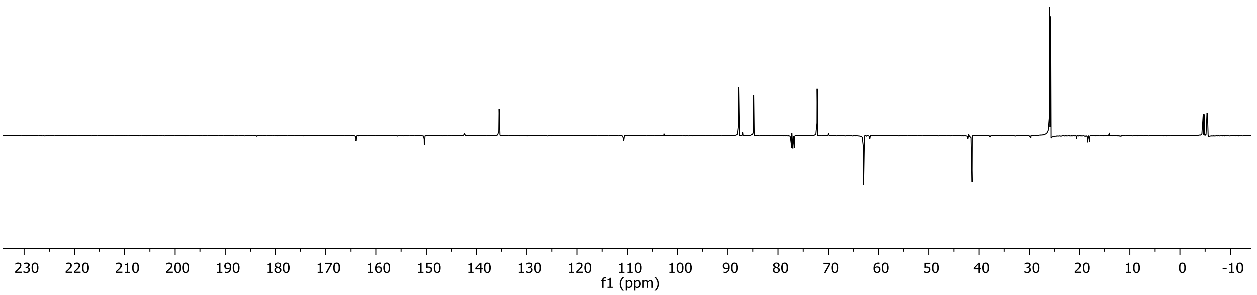

Figure S15: DEPTQ[2] NMR (100.67 MHz, $CDCl_3$, 25 °C) spectrum of compound **2**.

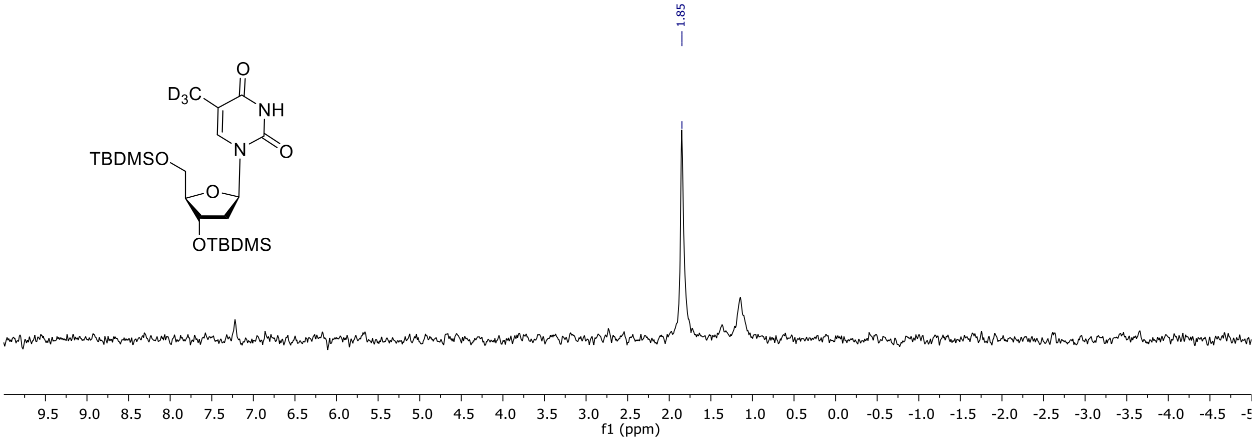

Figure S16: ^{2}H NMR (76.76 MHz, $CHCl_3$, 25 °C) spectrum of compound **2**.

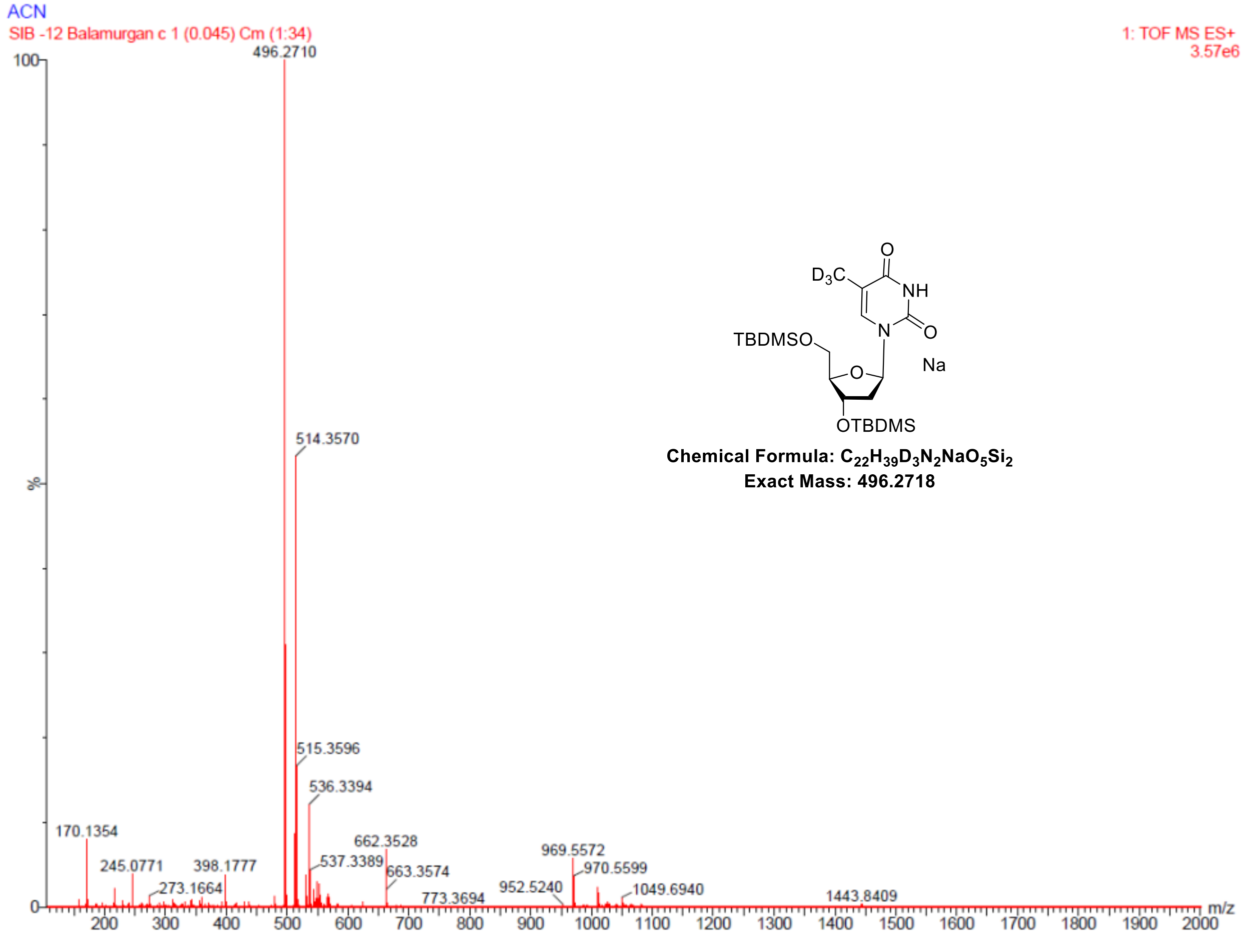

Figure S17: HRMS (ESI) spectrum of compound **2**.

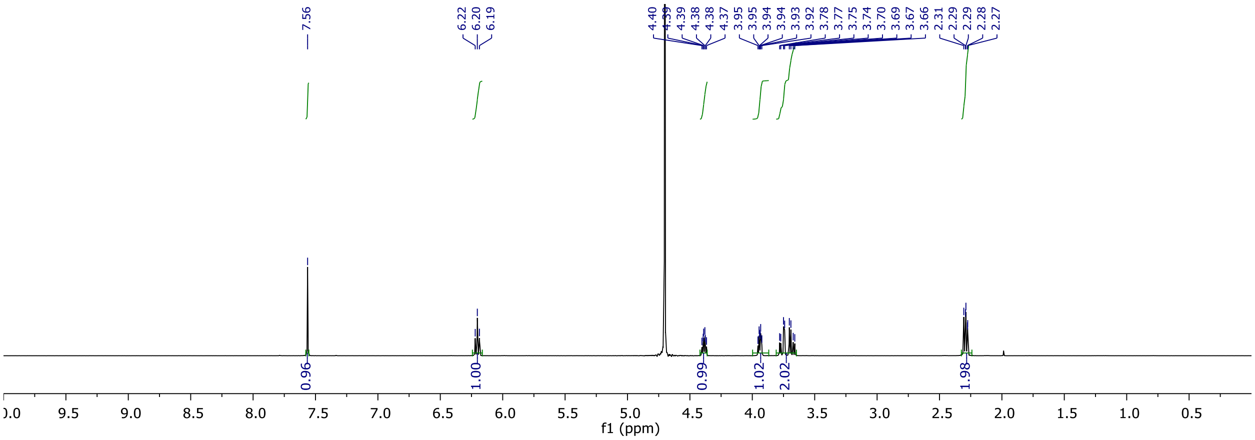

Figure S18: ^{1}H NMR (400.35 MHz, D_2O, 25 °C) spectrum of compound ***d*$_3$-thy**.

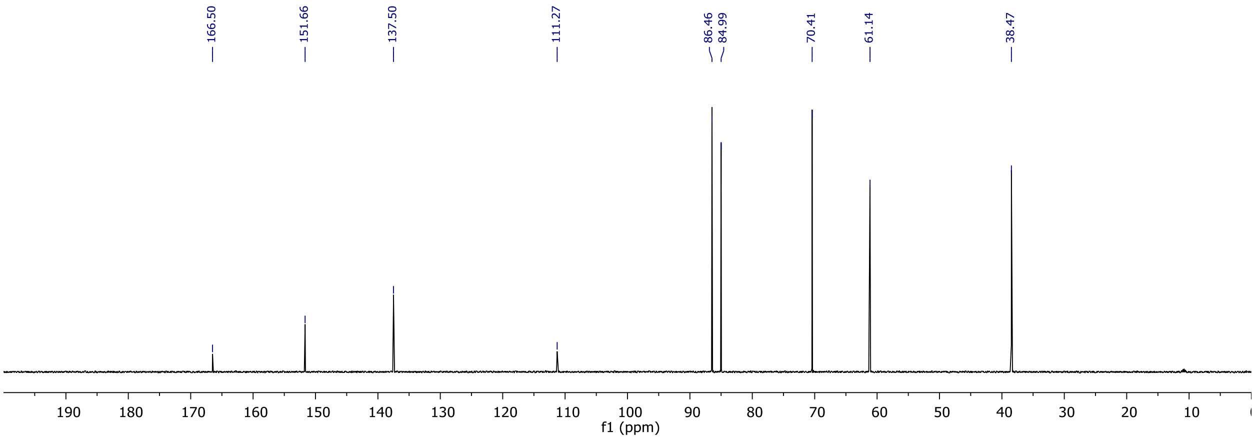

Figure S19: $^{13}C\{^{1}H\}$ NMR (100.67 MHz, D_2O, 25 °C) spectrum of compound ***d*$_3$-thy**.

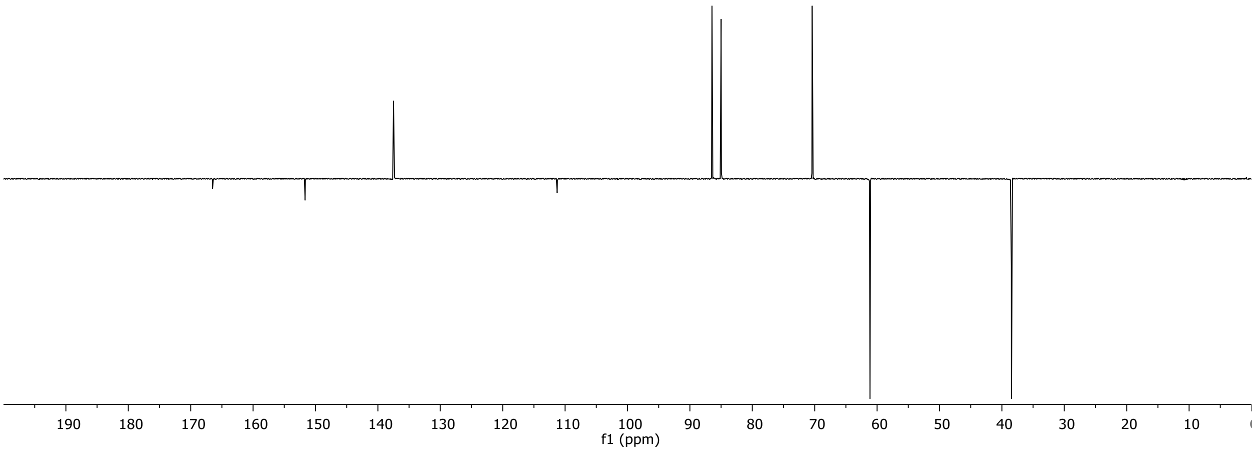

Figure S20: DEPTQ$\{^{1}H\}$ NMR (100.67 MHz, D_2O, 25 °C) spectrum of compound ***d*$_3$-thy**.

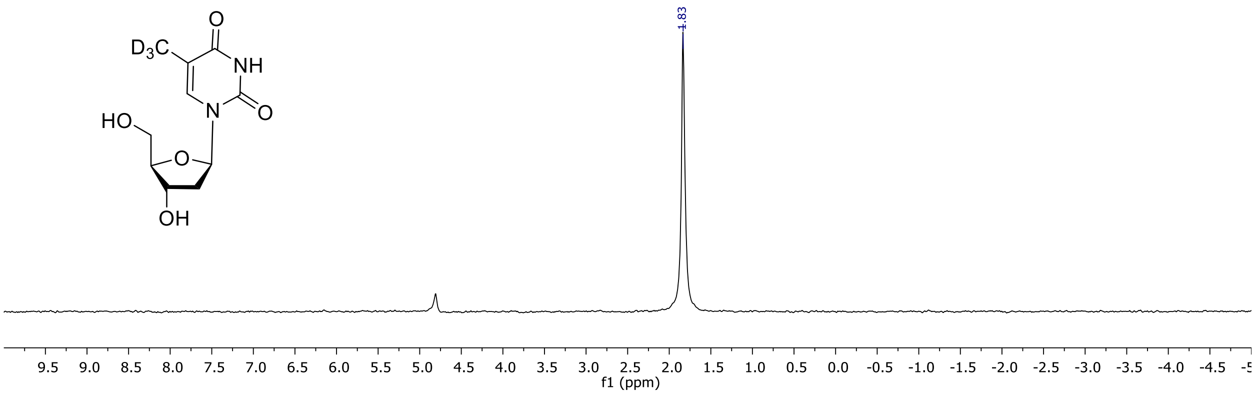

Figure S21: ^{2}H NMR (76.76 MHz, D_2O, 25 °C) spectrum of ***d*₃-thy**.

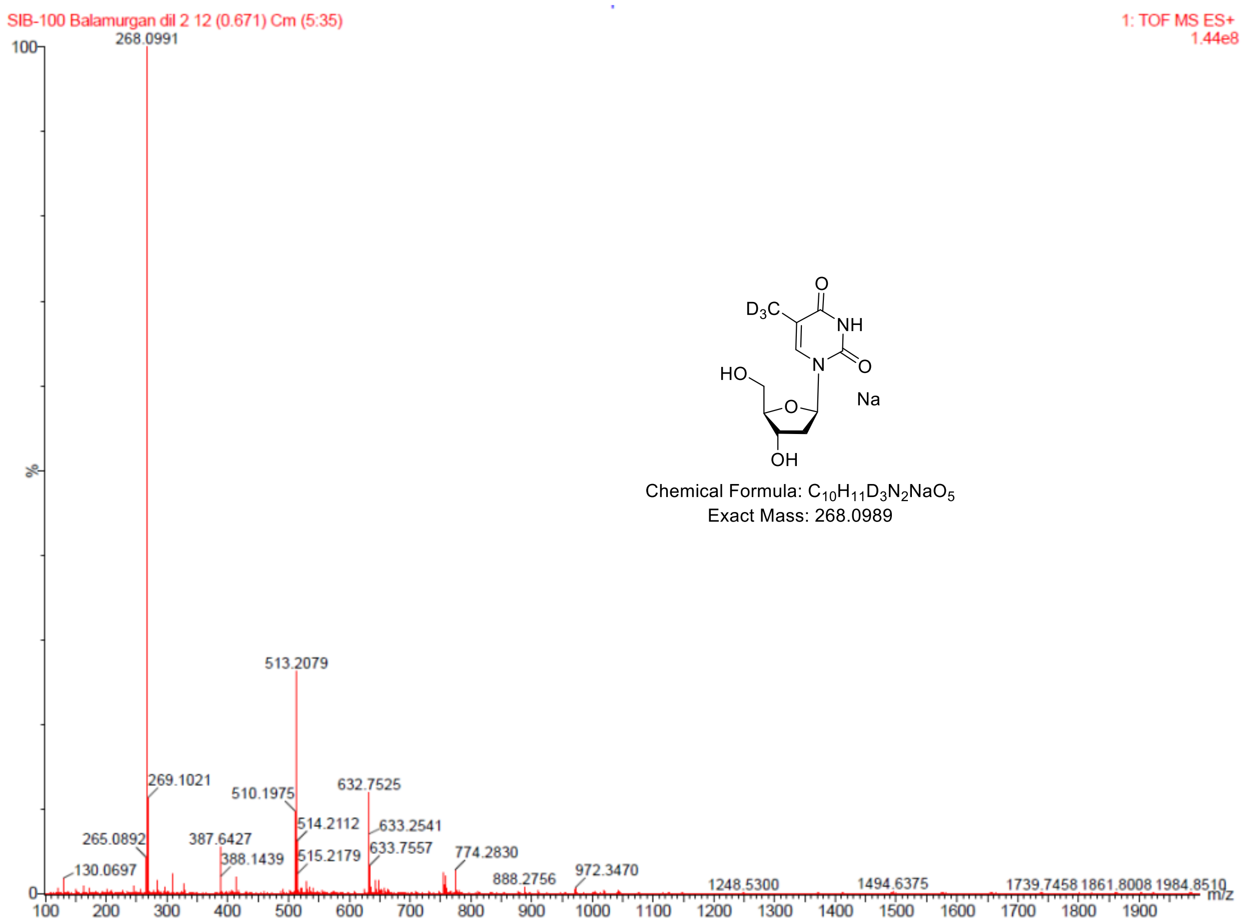

Figure S22: HRMS (ESI) spectrum of compound ***d*₃-thy**.

c. Supporting References

(1) Schneider, C. A.; Rasband, W. S.; Eliceiri, K. W. NIH Image to ImageJ: 25 years of image analysis. *Nat Methods* **2012**, 9 (7), 671-675.

(2) lLee, J. S. et al. Urea cycle dysregulation generates clinically relevant genomic and biochemical signatures. *Cell* **2018**, 174, 1559–1570 e1522.

(3) Hernando, D.; Kellman P.; Haldar J.P.; Liang ZP. Robust water/fat separation in the presence of large field inhomogeneities using a graph cut algorithm. *Magn Reson Med* **2010**, 63(1), 79-90.

(4) Peters, D. C.; Markovic, S.; Bao, Q.; Preise, D.; Sasson, K.; Agemy, L.; Scherz, A.; Frydman, L., Improving deuterium metabolic imaging (DMI) signal-to-noise ratio by spectroscopic multi-echo bSSFP: A pancreatic cancer investigation. *Magn Reson Med* **2021**, 86 (5), 2604-2617.
